# The *Entamoeba histolytica* Vps26 (EhVps26) retromeric protein is involved in phagocytosis: Bioinformatic and experimental approaches

**DOI:** 10.1371/journal.pone.0304842

**Published:** 2024-08-08

**Authors:** Diana Martínez-Valencia, Cecilia Bañuelos, Guillermina García-Rivera, Daniel Talamás-Lara, Esther Orozco

**Affiliations:** 1 Departamento de Infectómica y Patogénesis Molecular, Centro de Investigación y de Estudios Avanzados del Instituto Politécnico Nacional (Cinvestav), Ciudad de México, México; 2 Doctorado Transdisciplinario en Desarrollo Científico y Tecnológico para la Sociedad, Cinvestav, Ciudad de México, México; 3 Laboratorios Nacionales de Servicios Experimentales (LaNSE), Cinvestav, Unidad de Microscopía Electrónica, Ciudad de México, México; Cambridge University, UNITED KINGDOM OF GREAT BRITAIN AND NORTHERN IRELAND

## Abstract

The retromer is a cellular structure that recruits and recycles proteins inside the cell. In mammalian and yeast, the retromer components have been widely studied, but very little in parasites. In yeast, it is formed by a SNX-BAR membrane remodeling heterodimer and the cargo selecting complex (CSC), composed by three proteins. One of them, the Vps26 protein, possesses a flexible and intrinsically disordered region (IDR), that facilitates interactions with other proteins and contributes to the retromer binding to the endosomal membrane. In *Entamoeba histolytica*, the protozoan parasite responsible for human amoebiasis, the retromer actively participates during the high mobility and phagocytosis of trophozoites, but the molecular details in these events, are almost unknown. Here, we studied the EhVps26 role in phagocytosis. Bioinformatic analyses of EhVps26 revealed a typical arrestin folding structure of the protein, and a long and charged IDR, as described in other systems. EhVps26 molecular dynamics simulations (MDS) allowed us to predict binding pockets for EhVps35, EhSNX3, and a PX domain-containing protein; these pockets were disorganized in a EhVps26 truncated version lacking the IDR. The AlphaFold2 software predicted the interaction of EhVps26 with EhVps35, EhVps29 and EhSNX3, in a model similar to the reported mammalian crystals. By confocal and transmission electron microscopy, EhVps26 was found in the trophozoites plasma membrane, cytosol, endosomes, and Golgi-like apparatus. During phagocytosis, it followed the erythrocytes pathway, probably participating in cargoes selection and recycling. *Ehvps26* gene knocking down evidenced that the EhVps26 protein is necessary for efficient phagocytosis.

## Introduction

The retromer is a highly conserved eukaryotic protein complex, whose main function is the selection and transport of transmembrane cargo proteins, from early endosomes to plasma membrane, or from late endosomes towards the trans-Golgi network (TGN), maintaining the cellular homeostasis [1]. The retromer is a Rab7 effector [2, 3], and cargoes include the cation-independent mannose-6-phosphate receptor (CI-M6PR) [4, 5], the Wntless chaperone [6–11], the Alzheimer-associated protein SorLA [12], the GLUT1 glucose transporter [13, 14], among others. On the other hand, the retromer is a target for intracellular pathogens. It facilitates the transport and replication of viruses such as SARS-CoV-2, HIV, and HPV [15–17]; in the *Legionella pneumophila* bacteria, the RidL effector protein mimics a retromer component, allowing bacterial intracellular growth [18]; and in the *Toxoplasma gondii* protozoan, the retromer regulates the location of virulence factors [19], thus constituting a key player in pathogens virulence.

The yeast pentameric retromer is formed by a SNX-BAR membrane-remodeling heterodimer and the cargo-selecting complex (CSC). Instead, the mammalian SNX-BAR membrane-deforming heterodimer is composed by either SNX1 or SNX2 with SNX5 or SNX6 sorting nexin proteins (Vps5 and Vps17 in yeast). The main function of yeast Vps5/Vps17 proteins is the induction and stabilization of tubular regions in the endosomal membrane [20]. Nevertheless, the evidence in metazoan proteins suggests that SNX-BAR dimers can directly recognize and deliver cargo, beyond the retromer association, as in ATG9A trafficking by the SNX4-SNX7 dimer [21], the delivery of CI-MPR by SNX1/2 and SNX5/6, and other receptors [22–26]. The CSC includes the vacuolar protein sorting (Vps) Vps26, Vps35, and Vps29 proteins. Vps35 and Vps26 recognize the cytosolic domains of cargo molecules, and Vps35 is a scaffold protein for Vps26 and Vps29 assembly at the Vps35-N and -C terminal, respectively [27]. Vps29 joins to the TBC1D5 Rab7 regulator and VARP, a Rab21 GEF and Rab32 effector, promoting the retromer detachment from the endosomal membrane [28–30]. Vps26 participates in cargo transport and is the most conserved retromeric protein among eukaryotic lineages [31]. The *Vps26* gene deletion results in a decrease in Vps35 and Vps29 protein expression, and in Golgi apparatus fragmentation [5]. Furthermore, it could be lethal for murine embryos [32].

Vps26 proteins belong to the arrestin superfamily due to their structural identity [32, 33]. Three Vps26 variants have been found in higher eukaryotes: Vps26A and Vps26B in the retromer, and Vps26C in the retriever, a retromer-like complex involved in the recycling of proteins from endosomes towards the plasma membrane [34]. Vps26A and Vps26B differ in their C-terminal region, which binds different proteins [32, 35, 36]. Moreover, there, it is present an intrinsically disordered region (IDR) [32, 33, 37], whose amino acids provide hydrophobic segments that mediate cooperative folding. IDRs do not form secondary structures, but allow dynamic and reversible complexes formation [38]. They selectively bind to different ligands during transcription regulation, DNA repair, apoptosis, enzyme regulation, protein sorting, proteasomal degradation, chromatin remodeling [39–42], among others. In protozoa and bacteria, IDRs have been involved in survival, adaptation, and virulence [43].

In human cells, Vps26 and Vps35 interact with the Rab7 GTPase [3, 44], meanwhile, in yeast and plants, the Rab7 interaction occurs through Vps35 [45, 46]. In *Entamoeba histolytica*, the causative agent of human amoebiasis [47], the Vps26 protein (EhVps26) has a long carboxy-terminal, which contains the interaction site with Rab7A [48]. According to Nakada-Tsukui et al. (2005), this association, that carries out in a GTP-dependent manner, is important for the trophozoites cytotoxic effect caused by the cysteine-protease 2 [48]. In addition, EhVps26 interacts with two EhSNX3-like proteins [49], and EhVps35 [48]. Recently, our group has experimentally probed that EhVps35 is expressed and recycles virulence factors [50].

To deepen the *E*. *histolytica* retromer characterization, we performed here *in silico* analyses of retromer proteins from several eukaryotes. Then, we characterized the EhVps26 protein using *in silico* and experimental approaches. Molecular dynamics simulations (MDS) evidenced the EhVps26 characteristic arrestin folding. A truncated EhVps26 (EhVps26-t) version, lacking the C-terminal, revealed that the IDR confers stability and flexibility to EhVps26, probably providing versatility for the association of multiple ligands. The AlphaFold2 software predicted that EhVps26 binds to EhVps35, EhVps29 and EhSNX3. We then tracked the EhVps26 participation during phagocytosis. The protein was located in the plasma membrane, endosomes, phagosomes and Golgi-like structures. In addition, the *Ehvps26* gene knockdown evidenced that EhVps26 participates from early to late stages of phagocytosis. Overall, our results point out the potential relevance of EhVps26 in the binding of different ligands or counterparts during *E*. *histolytica* phagocytosis.

## Results

### 1.1 The *E*. *histolytica* retromer is formed by EhVps26, EhVps35, EhVps29, and EhSNX3 proteins

**Fig 1:**
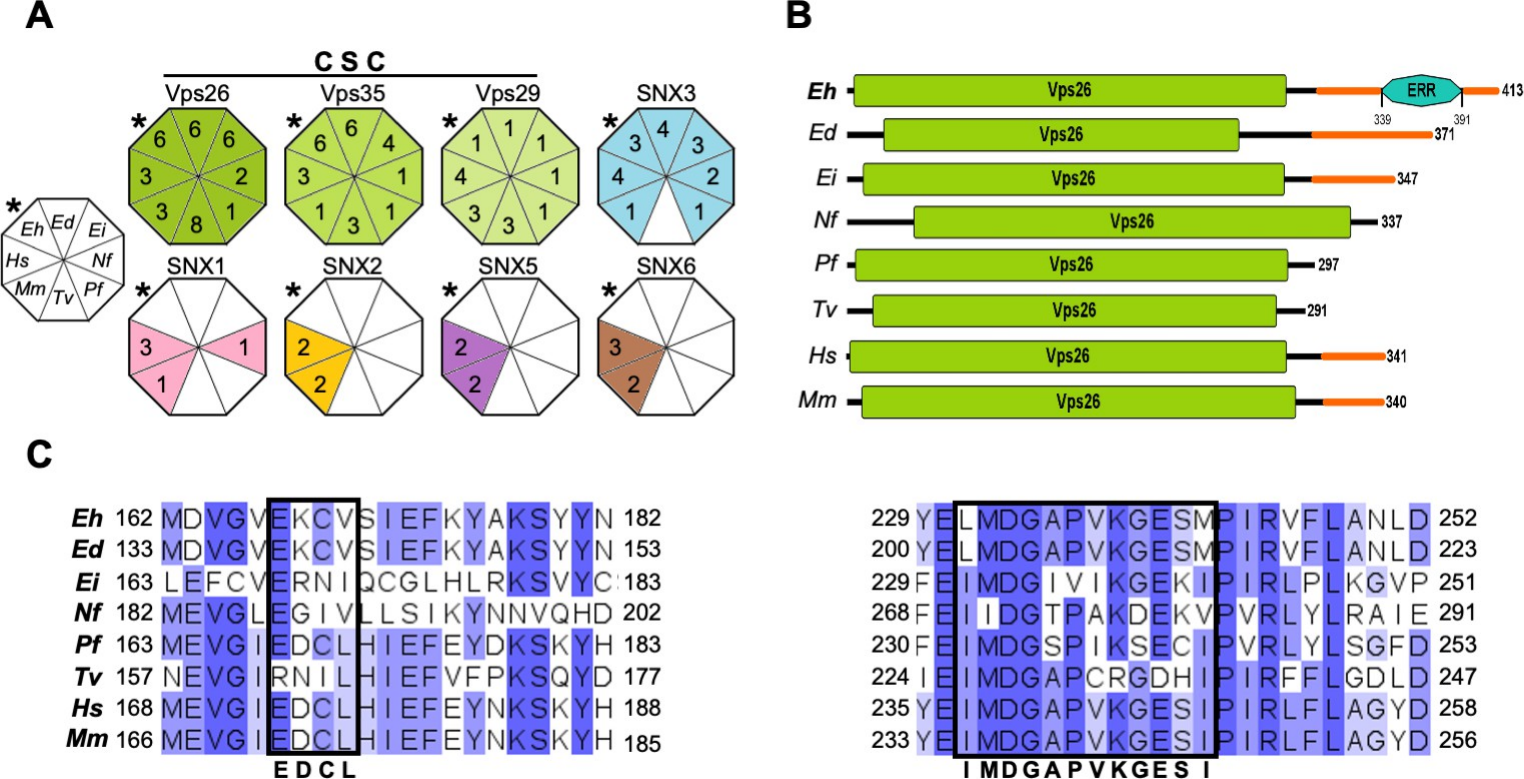
*In silico* comparison of retromer complexes and Vps26 proteins in different species. **A**. CSC: cargo selecting complex. Diagrams of the retromeric proteins in different species. The colored triangles and numbers inside them point out the distinct retromeric components in the selected species. The uncolored octagon at left indicates the species (Eh: *E*. *histolytica*, Ed: *E*. *dispar*, Ei: *E*. *invadens*, Nf: *N*. *fowleri*, Pf: *P*. *falciparum*, Tv: *T*. *vaginalis*, Mm: *M*. *musculus* and, Hs: *H*. *sapiens*). * indicate *E*. *histolytica*. Analyses considered isoforms reported in databases. **B.** Vps26 proteins diagrams. Vps26 domains are shown in green and IDRs in orange. Numbers at right: Proteins length. ERR: Glutamic acid rich region. **C.** Multiple alignment of Vps26 protein motifs. Darker blue: The most conserved residues among species. Black squares: Amino acids that interact with Vps35 and SNX3. Consensus motifs are indicated at the bottom. Numbers: Residues location.

In yeast and mammalian cells, the retromer regulates the retrieval of cargo proteins from the endocytic system to the TGN by retrograde pathways [51], or towards the plasma membrane using the recycling pathway [52]. The CSC recognizes the endosomal membrane-bound proteins, whereas the SNX membrane-remodeling complex helps the CSC to bind and curve the endosomal membrane [53]. Despite the current understanding of the retromer functions in higher eukaryotes, its role is poorly known in protozoa. As the aim of our work is focused on *E*. *histolytica*, we first compared *in silico* the retromer configuration of several amoebic species and protozoa of medical importance, using human and mouse Vps26, Vps35, Vps29, SNX1-SNX6 protein sequences as reference. According to the results displayed by the NCBI and VEuPathDB servers, all analyzed species showed all CSC components, although the number of molecules for each component varied (Fig 1A). The SNX membrane-remodeling complex also presented variations in the number of members among distinct species. However, we did not identify any SNX protein in *Trichomonas vaginalis*. Interestingly, the *Entamoeba* species did not exhibit PX-BAR proteins (SNX1, SNX2, SNX5, and SNX6), but they presented at least one SNX3 (Fig 1A). The considered protozoa did not show any SNX-BAR protein, with exception of *N*. *fowleri*, which exhibited one SNX-1 like protein. The relative simplicity in the protozoan retromers may be related to their antique origin. Moreover, previous works have reported that the retromer formed by CSC and at least one SNX3 protein is functional and capable of forming tubular regions in the endosomal membrane [11, 53, 54]. Analyses included all the sequences reported in databases, including isoforms and non-experimentally proved proteins.

### 1.2 EhVps26 is a *bona fide* Vps26 protein, but displays particular features

Vps26 proteins have a role in CSC assembly and cargo recognition [44, 55]. Here, we used the human Vps26 protein sequence as a reference to analyze the protein domains in different organisms. All analyzed Vps26 proteins displayed the canonical long Vps26 domain at their N-terminal, and an IDR at their C-terminal (Fig 1B). Remarkably, EhVps26 presented the longest C-terminal that includes an IDR enriched in Glu and Lys residues (Fig 1B). This highly charged region provides a flexible area to EhVps26 for molecules binding.

To continue with the EhVps26 structural characterization, we performed a multiple alignment to compare the EhVps26 sequence with putative orthologues. Our data evidenced that EhVps26 shares homology with the Vps26 proteins of human (45%), *T*. *vaginalis* (35%), *P*. *falciparum* (42%), and several amoebic species (33–87%) (S1 Table). In the canonical retromer, Vps26 binds to the Vps35 N-terminal, whereas Vps29 binds to the Vps35 C-terminal [27]. Hence, we searched for the 173-EDCL-176 and 237-IMDGAPVKGESI-248 motifs, that allow the interaction of Vps26 with Vps35. These regions are also involved in SNX3 binding [27, 33]. The *E*. *histolytica* and *E*. *dispar* Vps26 sequences displayed a substitution of Asp by Lys and a compensatory substitution of Leu by Val, resulting in the EKCV motif (Fig 1C). In addition, the 237-IMDGAPVKGESI-248 motif was found as LMDGAPVKGESM in EhVps26, where the underlined Leu231 and Met242 are compensatory exchangeable of Iso237 and Iso248. The *E*. *invadens* and *Naegleria fowleri* Vps26 proteins also exhibited substitutions in both motifs. Our findings predicted that Vps26 proteins and their functional domains are conserved in all analyzed species. They also suggest that EhVps26 contains the Vps26 domain and the motifs required for EhVps35 and EhSNX3 interaction. Altogether, our *in silico* results strongly suggest that the *E*. *histolytica* retromer is formed by CSC-SNX3 proteins. However, the role of the long IDR located in the EhVps26 C-terminal is still to be further studied.

### 1.3 The EhVps26 three-dimensional structure displays a conserved arrestin folding

**Fig 2:**
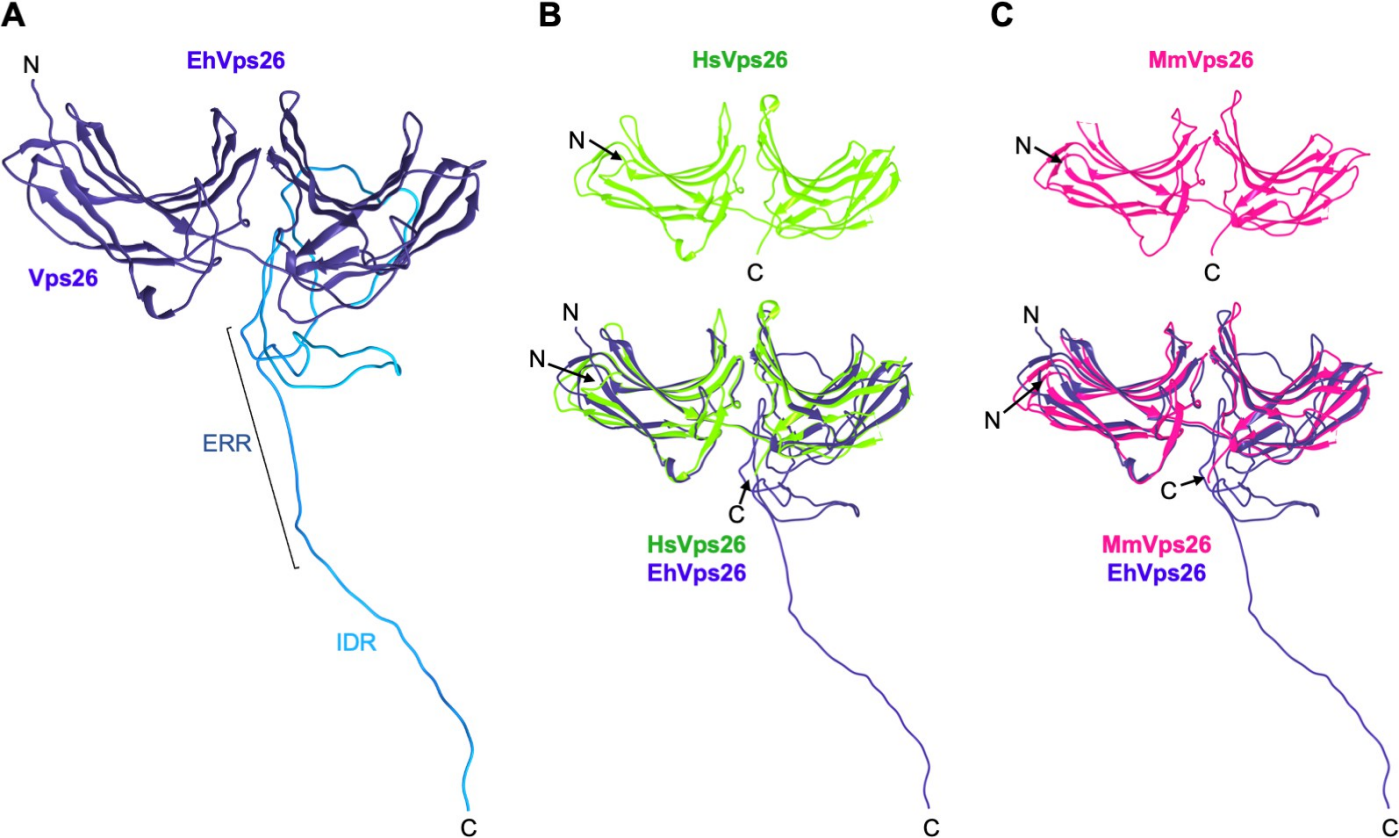
EhVps26 tridimensional structure. **A.** Best scored predicted structure obtained through the Modeller software. The IDR is shown in clear blue, indicating the ERR. **B, C.** Top: Human and mouse Vps26 crystal models. Bottom: Alignment of human and mouse Vps26 crystal models with EhVps26. RMSD: 2.1 and 2.3 Å, respectively. N and C: Protein terminus. Images were obtained by the Chimera software.

Vps26 proteins belong to the arrestin superfamily [32, 33], whose members are composed by two subdomains formed by 9 anti-parallel β-sheets each one, joined by a loop [56], as found in the human and mouse Vps26 crystals (PDB IDs: 2FAU and 2R51). Considering the presence of a long C-terminal in EhVps26, we used several servers based on different algorithms to generate its three dimensional (3D) structure and then, we chose the best-scored model. According to the Ramachandran plot values (S1 Fig), the Modeller software gave the top results, which were submitted to the GalaxyWEB server for refinement. Outcomes from these analyses showed 94.4 and 98.3% residues in favored and allowed regions, respectively (S1 Fig). The model showed a typical arrestin protein folding, while the IDR was modeled as a large loop (Fig 2A). This region could provide protein stability, interaction sites with other molecules, or additional functions not yet described. The EhVps26 3D model was then compared to the human and mouse Vps26 crystals (Fig 2B and 2C), and the root mean square deviation (RMSD) values were 2.1 and 2.3 Å, respectively. The high structural identity presented among EhVps26 and human and mouse Vps26 proteins reinforces its belonging to the Vps26 protein family. On the other hand, the presence of the IDR, and the high charge that it confers to the protein may be involved in the EhVps26 function [57].

### 1.4 The IDR confers structural stability to EhVps26

**Fig 3:**
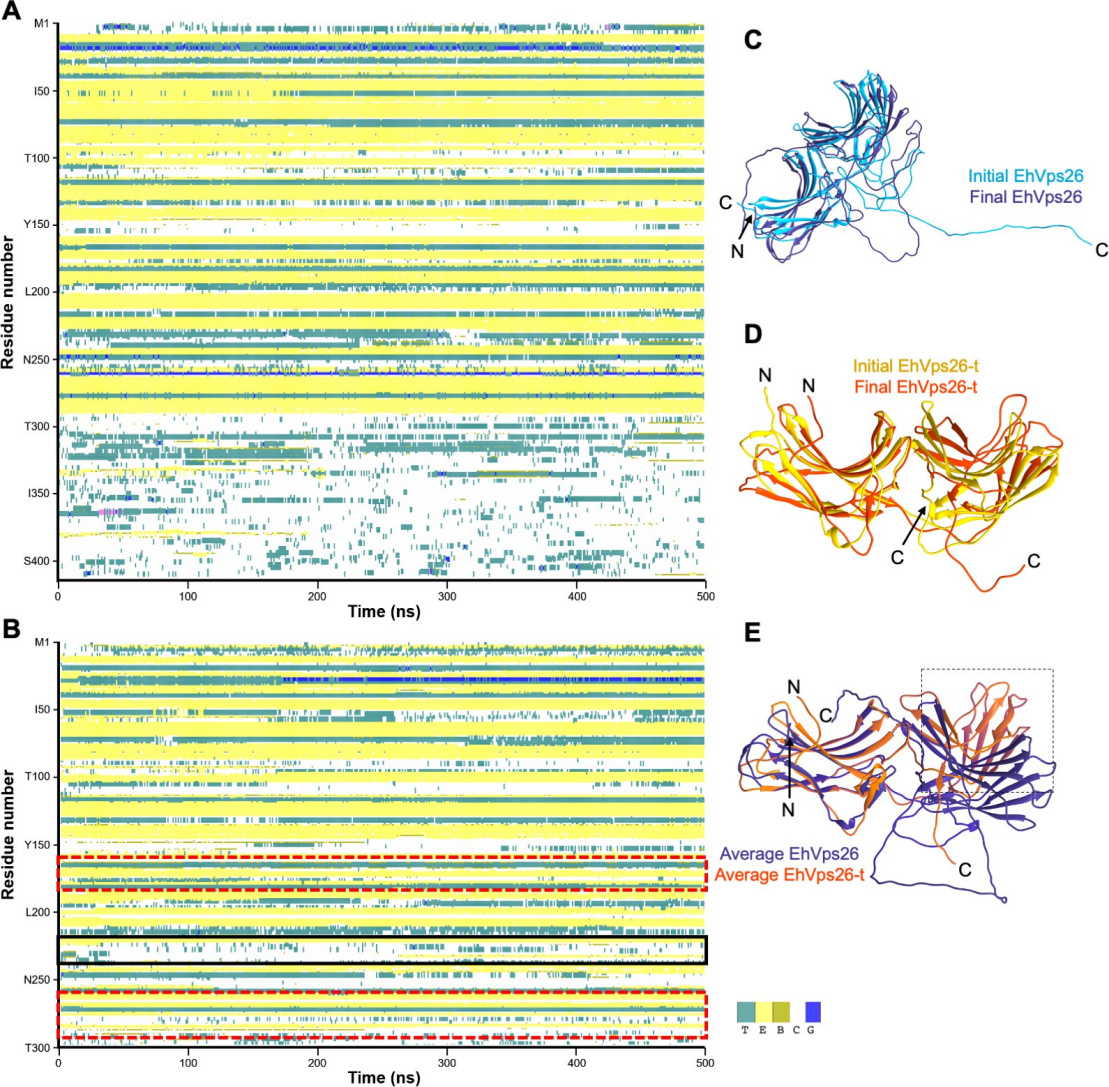
Structural changes of EhVps26 and EhVps26-t during MDS. **A.** EhVps26 secondary structure prediction. **B.** Secondary structure prediction of EhVps26-t. Red dotted-squares in B: areas of major conformational changes in β-sheets. Black square in B: Unstructured region for Vps35 and SNX3 interaction. Color codes: cyan-turn, yellow-β bulge, olive-β bridge, blue-310 helix, and white- coils. **C.** Initial (clear blue) and final (dark blue) EhVps26 structures after 500 ns MDS. **D.** Initial (yellow) and final (orange) EhVps26-t structures after 500 ns MDS. **E.** Overlapping of EhVps26 and EhVps26-t average equilibrated structures. Black-dotted square: Main regions affected in EhVps26-t. N and C: Protein terminus.

To further study the EhVps26 structural configuration and the role of its IDR, we generated a truncated version lacking the IDR (EhVps26-t), to compare both structures through 500 ns MDS in an aqueous environment. Then, the EhVps26 and EhVps26-t sequences were analyzed by the define secondary structure of proteins (DSSP) algorithm, which allows to determine the secondary structure of a given protein, based on its amino acid sequence [58]. The MDS results showed that EhVps26 and EhVps26-t conserved the two arrestin sub-domains formed by β sheets (Fig 3A, 3D). The EhVps26 IDR did not form any secondary structure during the MDS, which is a feature described for other IDRs under physiological conditions [40]. Meanwhile, the lack of IDR in EhVps26-t caused the shortening and re-location of several secondary structures (Table 1, Fig 3B and 3D). It is worth noting that the amino acids within the regions E167-V170 and E230-M242, involved in the EhVps35 and EhSNX3 putative interaction, formed coils instead of β-sheets in EhVps26-t (Fig 3E), pointing out the relevance of the IDR in providing the structural conformation needed for protein binding to other molecules, including EhVps35 and EhSNX3. The results described above suggest that the IDR contributes to the stability and location of secondary structures, influencing the protein dynamics and, hence, its function.

**Table 1:** β-sheets in EhVps26 and EhVps26-t after MDS.

β-sheet	EhVps26	EhVps26-t
1	Q10—L15	I11-L15
2	V24-K26	V24-I27
3	E34-M39	T33-M39
4	V44-T51	V44-T51
5	I62-E71	K63-E71
6	T79-N81	-
7	I83-S90	T79-S90
8	Y102-F106	K99-F106
9	R122-V131	R122-V131
10	L139-V147	L139-I148
11	I160-V164	I160-D163
12	S171-Y176	I172-Y176
13	V186-K194	G189-F193
14	S202-T213	Q208-T213
15	T220-E230	D222-V225
16	S241-F247	S241-I244
17	F264-E276	F264-Y268
18	K280-W291	-

### 1.5 The IDR influences the EhVps26 dynamics

**Fig 4:**
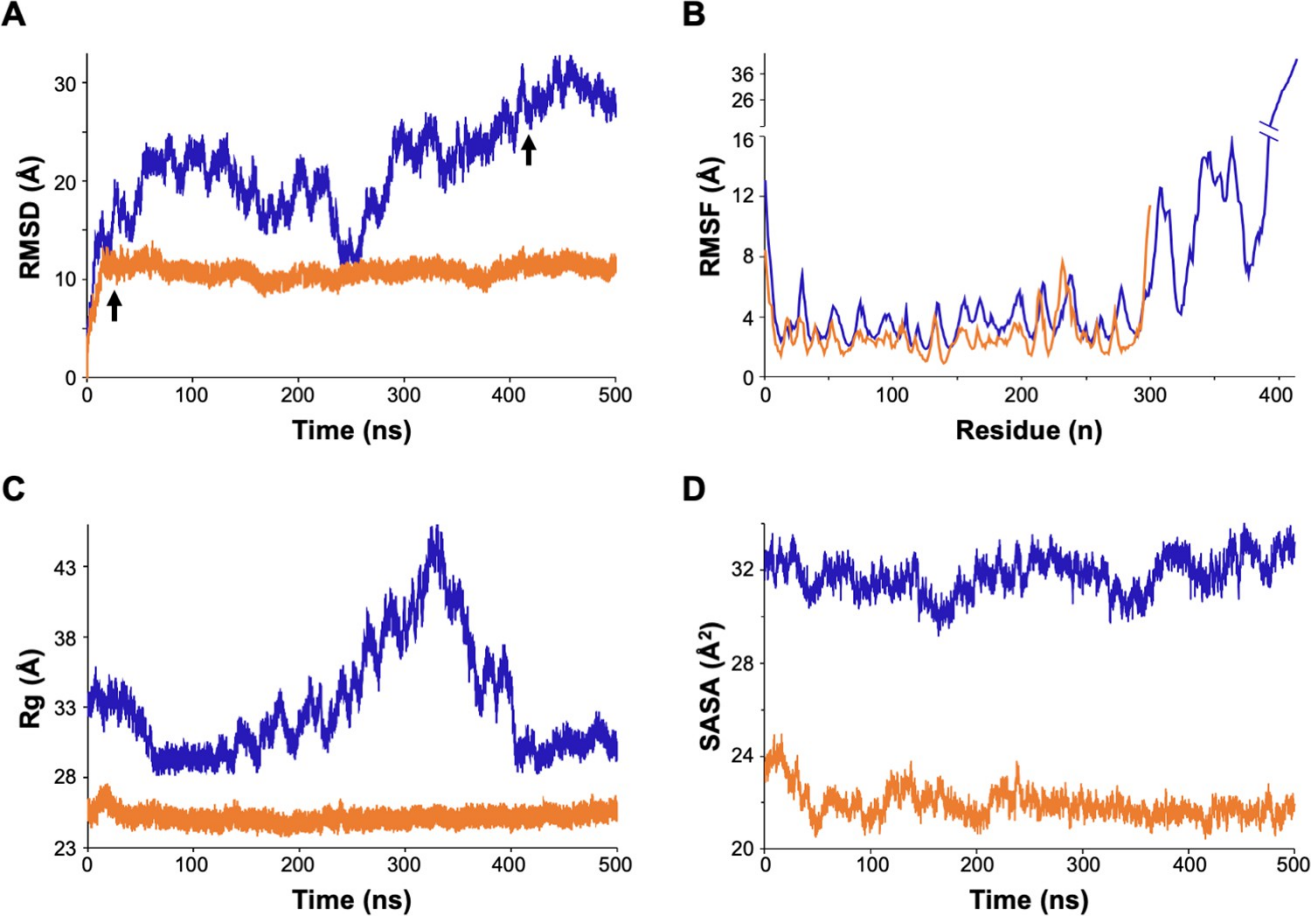
EhVps26 and EhVps26-t MDS analyses. **A.** RMSD for EhVps26 (blue) and EhVps26-t (orange) proteins. Arrows: Times for proteins stability. **B.** RMSF: Amino acids mobility of EhVps26 and EhVps26-t. **C.** Rg: Expansion of EhVps26 and EhVps26-t proteins. **D.** SASA: Solvent-accessible surface in EhVps26 and EhVps26-t proteins. For analyses, only the Cα backbone of each amino acid was considered.

To obtain additional insights regarding the influence of the IDR on molecular dynamics, we compared the EhVps26 and EhVps26-t RMSD trajectory, since this parameter determines the equilibration period and traces changes in the protein dynamics [59]. The values revealed that EhVps26 has high movement during the whole simulation. Fluctuation increased during the first 50 ns, diminished at 210 ns, and increased again at 280 ns, reaching stability at 410 ns (Fig 4A). Meanwhile, EhVps26-t presented a maximum RMSD value at 10 ns, then, the protein reached its stability in the first 20 ns, remaining stable until the end of the 500 ns MDS (Fig 4A). The root mean square fluctuation (RMSF) (a measure of the displacement of each residue, considering only the Cα), showed non-significant differences within the first 300 amino acids in both structures (Fig 4B). The most flexible area corresponded to the EhVps26 IDR (amino acids 300–413).

To determine the EhVps26 and EhVps26-t compactness, we measured the radius of gyration (Rg). EhVps26 presented maximum Rg values between 200 and 410 ns of simulation (Fig 4C). After this time, the Rg started to decrease and remained stable. In the case of EhVps26-t, the protein did not exhibit major changes during the whole MDS (Fig 4C). The Rg values correlated with the RMSD, confirming that EhVps26 and EhVps26-t reached stability after 410 and 20 ns, respectively.

Finally, we used the solvent-accessible surface area (SASA) parameter to determine the changes in the solvent accessibility of EhVps26 and EhVps26-t. The obtained values were between 30 and 33 Å for EhVps26 and, 21–25 Å for EhVps26-t (Fig 4D). The higher SASA value in EhVps26 may be explained by the high content of negative and positive amino acids within the IDR.

The results described above indicate that EhVps26 presents a higher flexibility, expansion, and accessible surface than EhVps26-t, features conferred by the IDR, which seems to be needed for EhVps26 interaction with other molecules.

### 1.6 EhVps26 displays pockets for the putative interaction with EhVps35, EhSNX3 and a PX domain-containing protein

**Fig 5:**
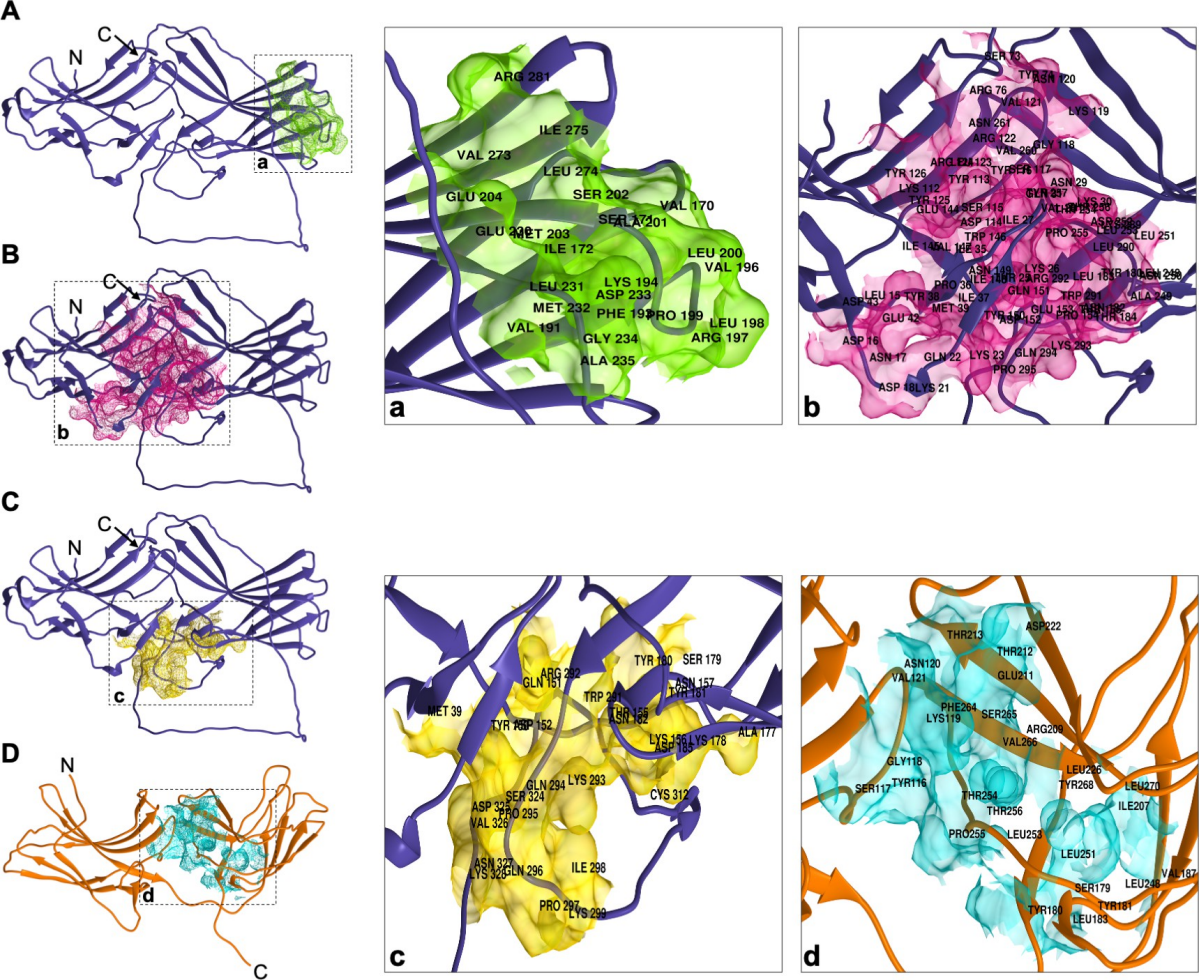
Binding pockets in EhVps26 and EhVps26-t. A,B,C. Localization of predicted binding pockets in the equilibrated EhVps26 structure. **D.** Predicted pocket in the EhVps26-t equilibrated structure. At right: Zoom of each pocket in black-dotted squares in A,B,C,D. Residues identity are indicated in each pocket.

The human Vps26 237-IMDGAPVKGESI-248 motif allows the interaction with Vps35 to form the retromer complex [33, 60]. We obtained an *in silico* EhVps26 equilibrated structure and analyzed its performance under simulated conditions. The DOGSiteScorer server predicted the binding sites to other molecules, including drug targets. We identified 15 putative binding pockets in EhVps26. Three of them, exhibited the highest *druggability* scores (cutoff 0.8), presenting large volumes, high depths, and high polar amino acids content (Table 2), which are the main criteria for determining the best potential binding sites in proteins [61]. These regions are exposed. The first pocket included the amino acids V170-L172, V191, F193, Y194, V196-E204, E230-A235, V273-I275 and R281 (Fig 5A). In human, these amino acids comprise the region for Vps35, SNX3 and the DMT1 cargo interaction [27, 36]. The second pocket corresponded to the amino acids M39, Y150-D152, T155-N157, A177-N182, D185, W291-Y299, C312 and S324-Y328; and the third pocket, close to the second one, was formed by the L15-D18, K21-K23, T25-N31, I35-M39, E42-D43, S73-Y74, R76, Y112-Y126, E144-T155, Y180, N182-T184, L248-Y257, V260-N261 and Y289-P295 residues (Fig 5B). The second and third pockets included the Y38, E42, K293, and P295amino acids, that in human Vps26A have been described as interaction sites for SNX27 [13, 62]. The results were reinforced by the PASSer server, which also predicted the second pocket as an allosteric site with a score of 53.63% (Fig 5B). Interestingly, the DOGSiteScorer server predicted 13 pockets in EhVps26-t, but only one reached the 0.8 cutoff (Table 2), considered as a confidence value. This pocket corresponded to the Y116-V121, S179-Y181, L183, V187, I207, R209, E211, T212-T213, D222, L226, L248, L251-T256, F264-V266, Y268 and L270 amino acids, for the binding of an unknown ligand (Fig 5C). However, the pocket is not exposed on the surface, suggesting that it is not functional.

**Table 2:** Features of EhVps26 and EhVps-t binding pockets.

	Pocket	Residues	Volume (Å^3^)	Surface (Å^2^)	Depth (Å)	*Druggability* score
EhVps26	1	V170-L172, V191, F193, Y194, V196-E204, E230-A235, V273-I275, R281	379.38	825.8	20.09	0.8
	2	M39, Y150-D152, T155-N157, A177-N182, D185, W291-Y299, C312, S324-Y328	631.35	1113.7	22.79	0.84
	3	L15-D18, K21-K23, T25-N31, I35-M39, E42, D43, S73, Y74, R76, Y112-Y126, E144-T155, Y180, N182-T184, L248-Y257, V260, N261, Y289-P295	2137.87	2696.9	24.30	0.8
EhVps26-t	1	Y116, S117, G118, K119, N120, V121, S179, Y180, Y181, L183, V187, I207, R209, E211, T212, T213, D222, L226, L248, L251, D252, L253, T254, P255, T256, F264, S265, V266, Y268, L270	486.46	791.01	22.69	0.84

Our results corroborated that the IDR stabilizes EhVps26 to allow its potential joining to other molecules, such as EhVps35 and EhSNX3. Although the third pocket in EhVps26 harbors a putative interaction site for a SNX27 PX-PDZ-FERM protein, in the *E*. *histolytica* genome we only found a 982 amino acids protein that contains a PX domain and a large loop, but does not share homology with SNX27 proteins, which have been reported as exclusive for metazoa [63].

### 1.7 *E*. *histolytica* CSC and CSC-SNX3 complexes mimic the mammalian ones

**Fig 6:**
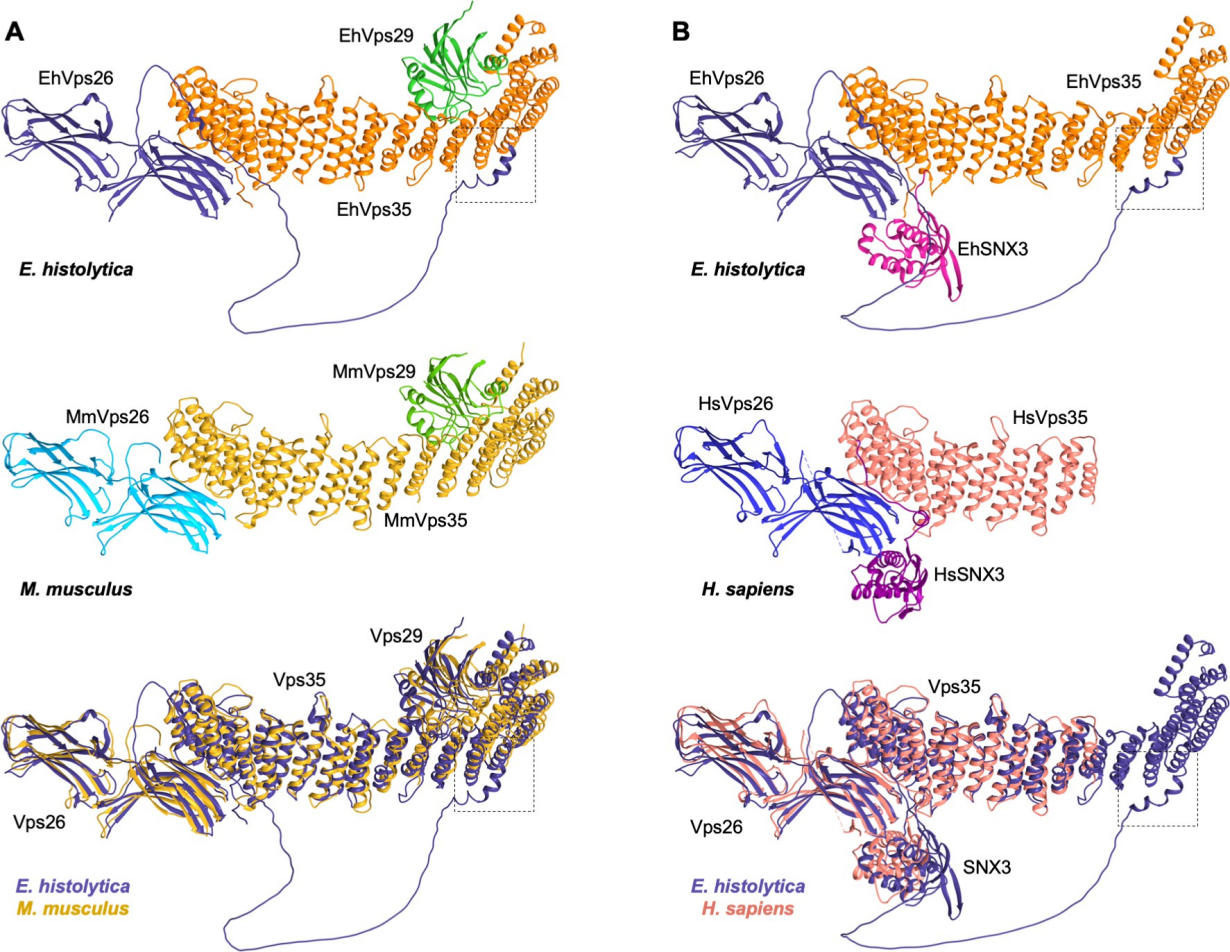
CSC and Vps26-Vps35-SNX3 complexes constitution predicted by the AlphaFold2 software. **A.** CSC predicted structures. Top: *E*. *histolytica*, middle: *M*. *musculus* CSC crystal model bottom: Overlapping structures. **B.** Vps26-Vps35-SNX3 predicted structures. Top: *E*. *histolytica*,: middle: *H*. *sapiens* Vps26-Vps35-SNX3 crystal model, bottom: Overlapping structures. Components are labeled and shown in different colors. Black-dotted squares: EhVps26 and EhVps35 interaction sites within the IDR.

Previous works have described the CSC structure in yeast and mammals [27, 64]. It is possible to predict protein and complexes structures, using powerful tools as AlphaFold2, an artificial intelligence system with atomic-level accuracy [65]. We used the AlphaFold2 multimer hosted in COSMIC^2^ [66], to obtain the *E*. *histolytica* CSC full structure. The results revealed that R3, R58, Y104, A106, D134, R140, A141, H144, N146, K147, R196, K552, K556 and K581 residues of EhVps35, and C169, E230, E204, R245, D233, E240, D405, D409, F401 and N411 residues of EhVps26 interact (Fig 6A, Table 3). It is worth noting that F401, D405, D409 and N411 EhVps26 residues are located within its IDR, comprising non-previously described interaction sites within the CSC (Fig 6A, black dotted squares and Table 3, shadowed squares).

**Table 3:** Interacting amino acids in CSC and EhVps26-EhVps35-EhSNX3.

*E*. *histolytica* CSC						*E*. *histolytica* EhVps26-EhVps35-EhSNX3					
EhVps35	EhVps26	Distance	EhVps35	EhVps29	Distance	EhSNX3	EhVps35	Distance	EhSNX3	EhVps26	Distance
R3	C169	2.75	R489	A16	2.87	Y4	N191	2.91	R122	R197	2.97
R58	E230	3.45*	R489	E44	2.69*	S2	Q247	2.18	E8	R197	3.19*
Y104	E230	3	R489	E44	2.85	Y4	Q247	2.86	E40	R197	2.55*
Y104	E204	3.3	K535	D144	3.23*	S2	Q247	2.65	E40	R197	2.9
A106	E230	2.91	K565	D62	2.87				Y7	A235	2.7
D134	R245	2.27*	Y569	D62	3.17						
R140	E240	3.81*	E604	K60	3.34*						
A141	D233	2.76	K656	D95	3.02*						
H144	D233	2.91	N696	R104	2.97						
N146	D233	2.62	N696	R104	3.01						
K147	D233	2.63*	N696	I101	3.26						
R196	E240	2.36*	N737	R104	3.02						
K552	D405	3.82*									
K552	D409	2.48*									
K556	F401	3.12									
K581	N411	3.21									

Shadowed amino acids, particular interactions in *E*. *histolytica*, within the IDR

Distance is represented in Å

* Interactions mediated by salt bridges

On the other hand, we found R489, K535, K565, Y569, E604, K656, N696 and N737 EhVps35 residues in interaction with A16, E44, D144, D62, K60, D95, R104, and I101 EhVps29 residues (Fig 6A, Table 3), in a similar way than the mouse CSC. Here, an interaction between N696 of EhVps35 and R104 of EhVps29 was found, with no equivalent interactions described in the mouse complex, which, once again, suggests specific sites for proteins association within the amoebic CSC structure. The RMSD between mouse and amoebic complexes was 1.4 Å.

We also used the human crystal containing Vps35, Vps26 and SNX3 proteins, to compare the corresponding complex in *E*. *histolytica*. We found that both models appear superimposed, strongly suggesting that the amoebic retromeric complex adopts a CSC-SNX3 configuration. Interacting residues for each amoebic component of this putative complex are shown in Table 3. We found less interactions in this model than in the human one. However, the EhVps26-EhSNX3 interaction has been proved *in vitro* [49]. The RMSD shared between Vps26-Vps35-SNX3 human and amoebic complexes was 1.9 Å.

The results described above confirmed that amoebic CSC and EhVps26-EhVps35-EhSNX3 complexes resemble the structures of mammalian orthologues, probably forming a functional *E*. *histolytica* CSC-SNX3 retromer.

### 1.8 EhVps26 is located in plasma membrane, cytosol, endosomes, and Golgi-like structures

**Fig 7:**
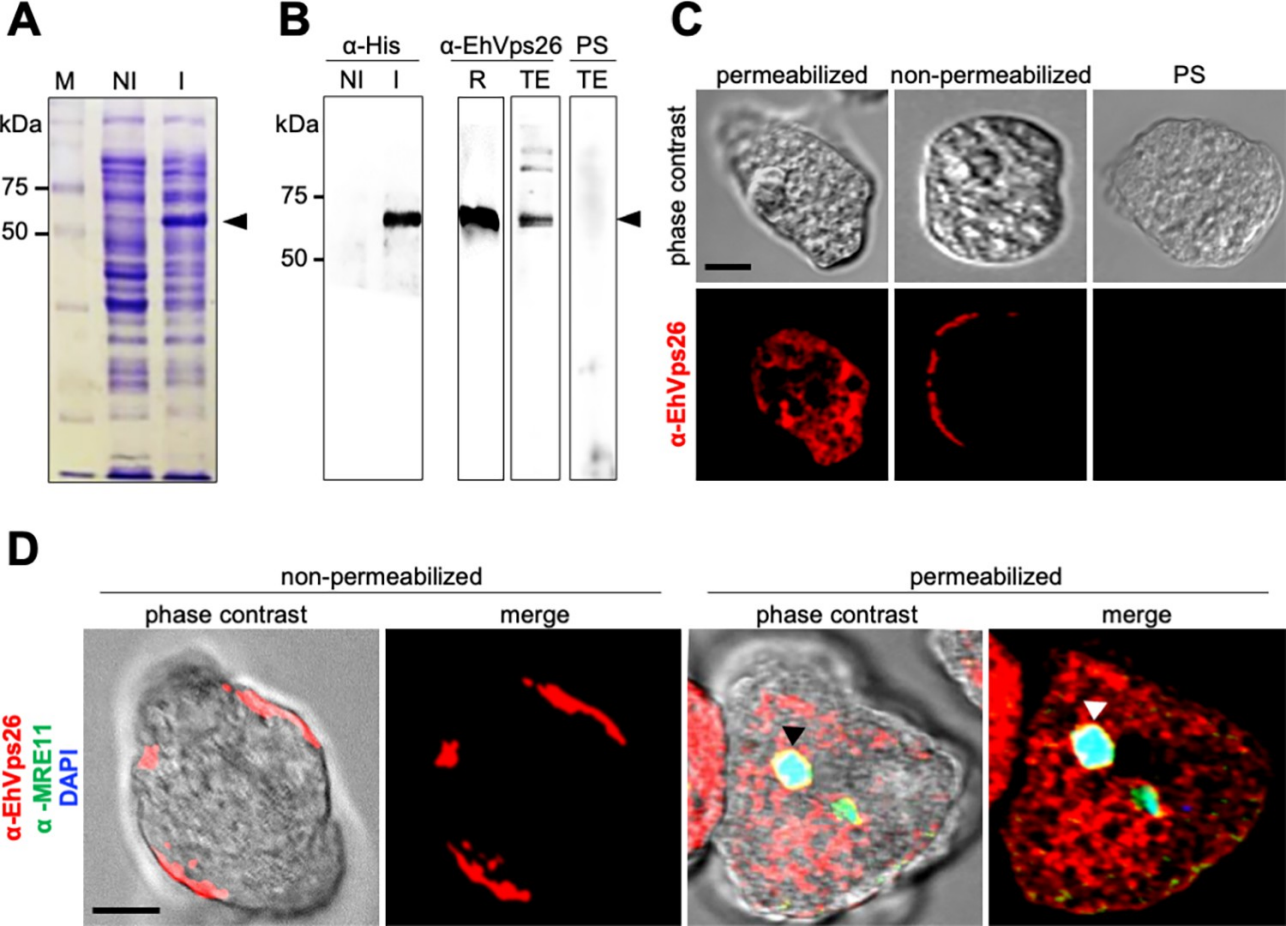
Expression and cellular location of EhVps26. **A.** Coomassie-stained gel with non-induced (NI) and induced (I) bacteria transformed with the pCold-*Ehvps26* plasmid. Arrowhead: EhVps26r. M: Molecular weight marker. **B.** Western blot using α-His and α-EhVps26 antibodies. R: Purified EhVps26r. TE: Total extracts *of E*. *histolytica*. PS: Preimmune serum. **C.** Confocal immunofluorescence of permeabilized and non-permeabilized fixed trophozoites treated with α-EhVps26 antibodies. PS: Preimmune serum. **D.** Confocal immunofluorescence of permeabilized and non-permeabilized fixed trophozoites treated with α-EhVps26 and α-MRE11 antibodies (control), and DAPI. Arrowhead: Nucleus.

In higher eukaryotes, the retromer localizes at the cytosol, the membrane of early endosomes, and the TGN during cargo recycling [2, 8, 10, 67]. In *E*. *histolytica*, EhVps35 has been also found in cytosol and endosomes [50]. In this work, we explored the EhVps26 location. First, we cloned the *Ehvps26* gene in the pCold I vector to generate a His-tagged recombinant protein (EhVps26r) (Fig 7A) and produced rabbit polyclonal antibodies against it (α-EhVps26). In transformed bacteria, the α-His tag antibodies detected EhVps26r with an estimated weight of 60 kDa, 58 kDa corresponding to EhVps26, and 2 kDa to the His-tag (Fig 7B). Meanwhile, the α-EhVps26 antibodies detected the bacterial purified recombinant protein and a 60 kDa band in amoebic lysates (Fig 7B). As expected, no bands were detected with the pre-immune serum (Fig 7B). Although the EhVps26 predicted molecular weight is 48 kDa, an aberrant migration could be caused by the high negative charge of the protein, as described for other EhVps proteins, including the ones belonging to the Endosomal Sorting Complexes Required for Transport [68]. Afterward, we performed confocal immunofluorescence assays using the α-EhVps26 and TRITC-labelled secondary antibodies on permeabilized and non-permeabilized amoebae (Fig 7C). In permeabilized trophozoites, EhVps26 was detected in the cytosol, as punctuated signals, probably corresponding to endosomal compartments. In non-permeabilized amoebae, EhVps26 was detected in the outer face of the plasma membrane, an atypical location for Vps26 proteins (Fig 7D). The pre-immune serum did not give any signal (Fig 7D). To verify plasma membrane integrity and α-EhVps26 specificity, we used a commercial antibody against MRE11 (Santa Cruz, USA), a conserved DNA-repairing enzyme, for immunofluorescence assays, using permeabilized and non-permeabilized trophozoites. Again, in non-permeabilized amoebae, EhVps26 was located at the plasma membrane, whereas no signal for EhMRE11 was detected there (Fig 7D), indicating that the antibody against MRE11 did not penetrate the plasma membrane. Accordingly, in permeabilized amoebae, EhVps26 was detected in cytosol. Meanwhile, EhMRE11 was found in the nucleus, as revealed by DAPI (Fig 7D).

**Fig 8:**
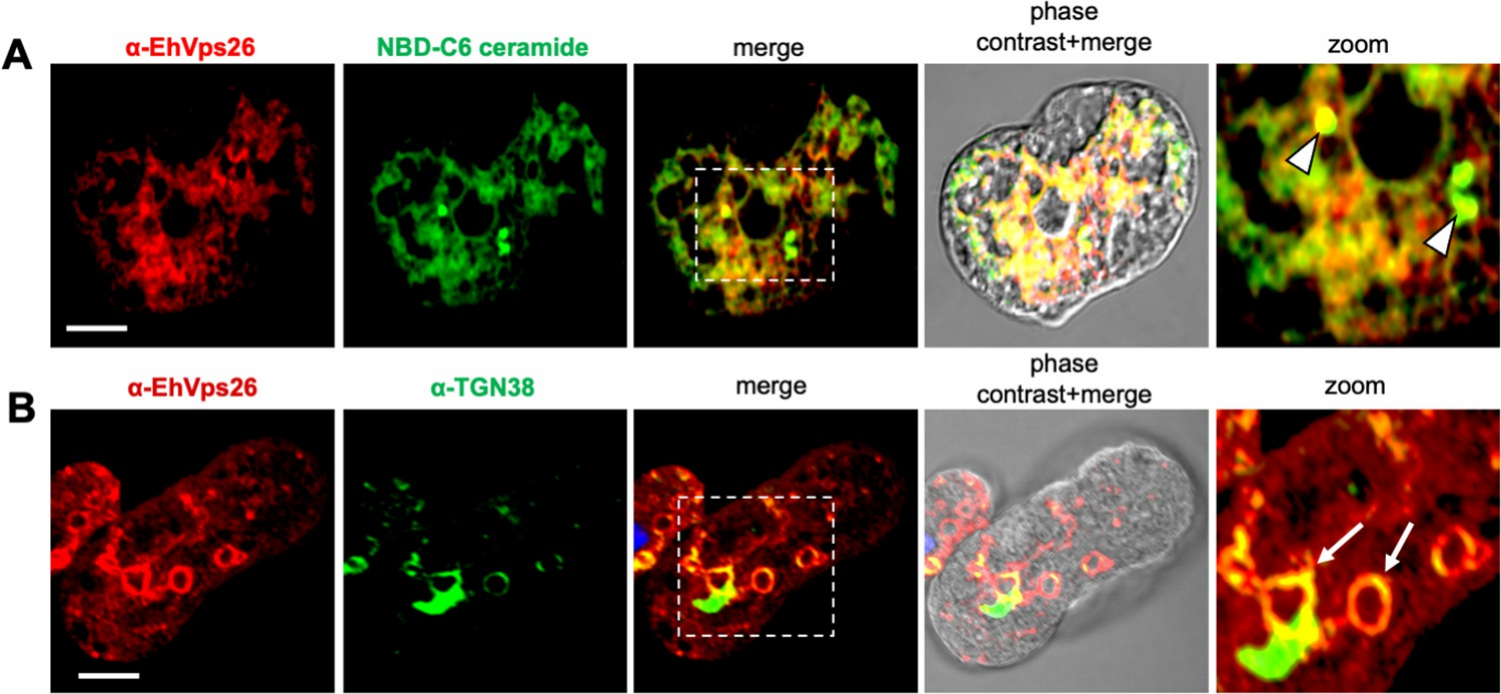
Localization of EhVps26 in Golgi-like structures. **A.** Confocal immunofluorscence assays using α-EhVps26 and the NBD-C6 ceramide molecular probe. Zoom of white-dotted square in the merged image. Arrowhead: EhVps26 colocalizing with Golgi -like structures. Scale bar: 10 μm. **B.** Confocal immunofluorescence assays using α-EhVps26 and α-TGN38. Arrows: Vesicles of EhVps26 colocalization with Golgi-like structures. Zoom of white-dotted square in the merged image. Scale bar: 10 μm.

Although a primitive Golgi apparatus has been described in *E*. *histolytica* as a membranous system [69–71], it remains unknown if the retromeric proteins are located at this organelle. Thus, we used the NBD-6C ceramide molecular probe and α-TGN38 antibodies as Golgi markers [71, 72]. The confocal images revealed α-EhVps26 and NBD-6C ceramide molecular probe co-localizing in Golgi-like structures (Fig 8A), with a Pearson’s coefficient of 0.77. Meanwhile, the α-TGN38 antibody, a marker of the Golgi *trans*-face, co-localized with the α-EhVps26 in membranous structures, including some around the nuclei (Fig 8B).

**Fig 9:**
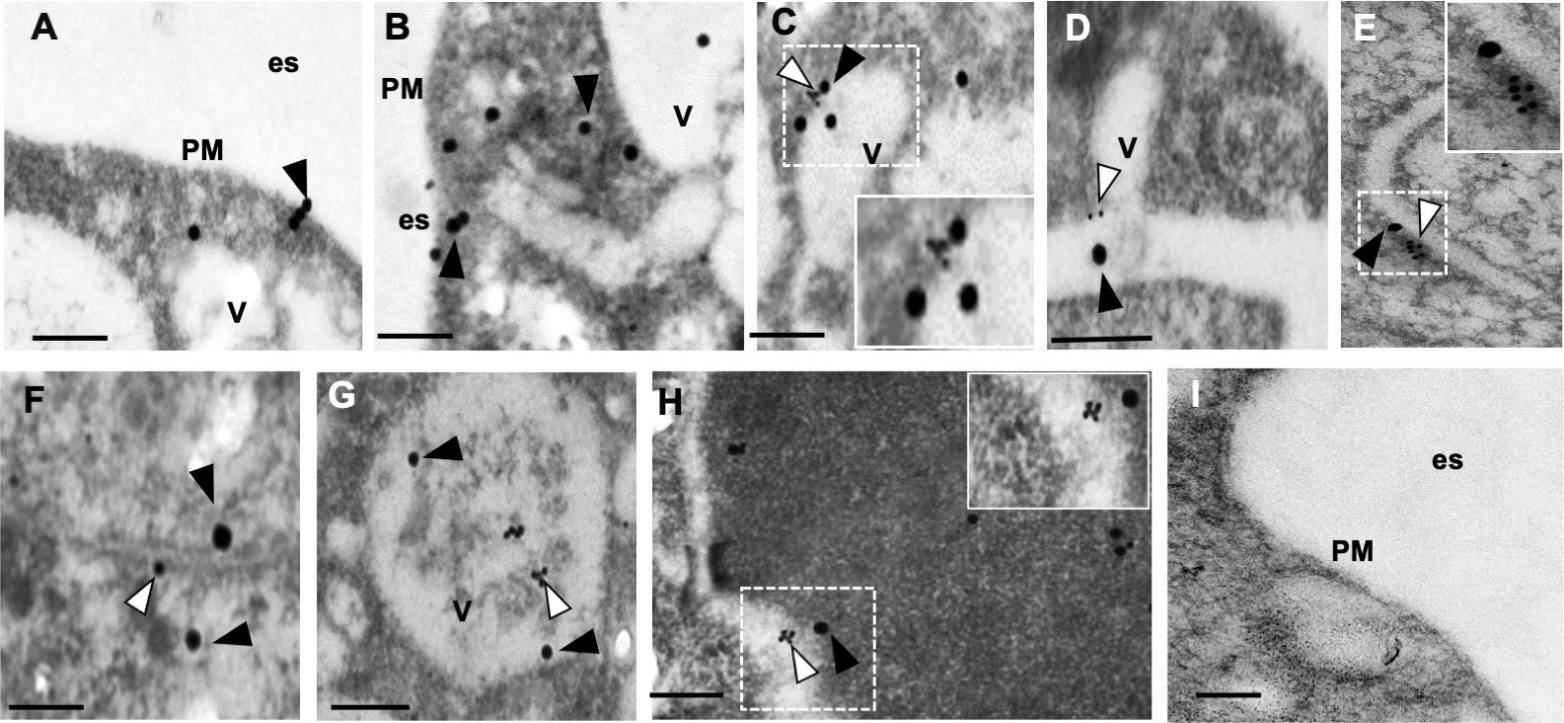
Ultrastructural localization of EhVps26 by TEM. Trophozoites thin sections were treated with rabbit α-EhVps26 and mouse α-TGN38 followed by 30 nm gold-labeled anti-rabbit and 15 nm gold-labeled anti-mouse secondary antibodies. **A,B.** Location of EhVps26 in plasma membrane, cytoplasm and vesicles. **C,D.** EhVps26 and EhTGN in tubular membranous structures. **E,F.** EhVps26 and EhTGN membranous cisternae-like structures. **G,H**. EhVps26 and EhTGN in different types of vesicles. **I.** Negative control. PM: plasma membrane. Es: Extracellular space. V: Vesicles. White arrowheads: EhTGN. Black arrowheads: EhVps26. Scale bar: 200 nm.

To gain insights on EhVps26 location in Golgi-like structures, we performed transmission electron microscopy (TEM), using α-TGN38 and α-EhVps26 antibodies, and gold-labeled secondary antibodies. TEM micrographs evidenced the presence of EhVps26 in the plasma membrane (Fig 9A, 9B) and near to EhTGN in vesicles (Fig 9C–9H) and membranous structures (Fig 9E and 9F). These last images resembles the typical Golgi apparatus in higher eukaryotes.

Altogether, these results show, for the first time, the location of EhVps26 in Golgi-like structures, by confocal microscopy and TEM assays, strengthening the role of EhVps26 in cargo recycling, as described in other eukaryotes [5]. Moreover, we recently have shown that EhVps35 participates in the recycling of the EhADH adhesin, and the Gal/GalNac lectin [50].

### 1.9 EhVps26 is mobilized during phagocytosis since the initial contact with target cells

**Fig 10:**
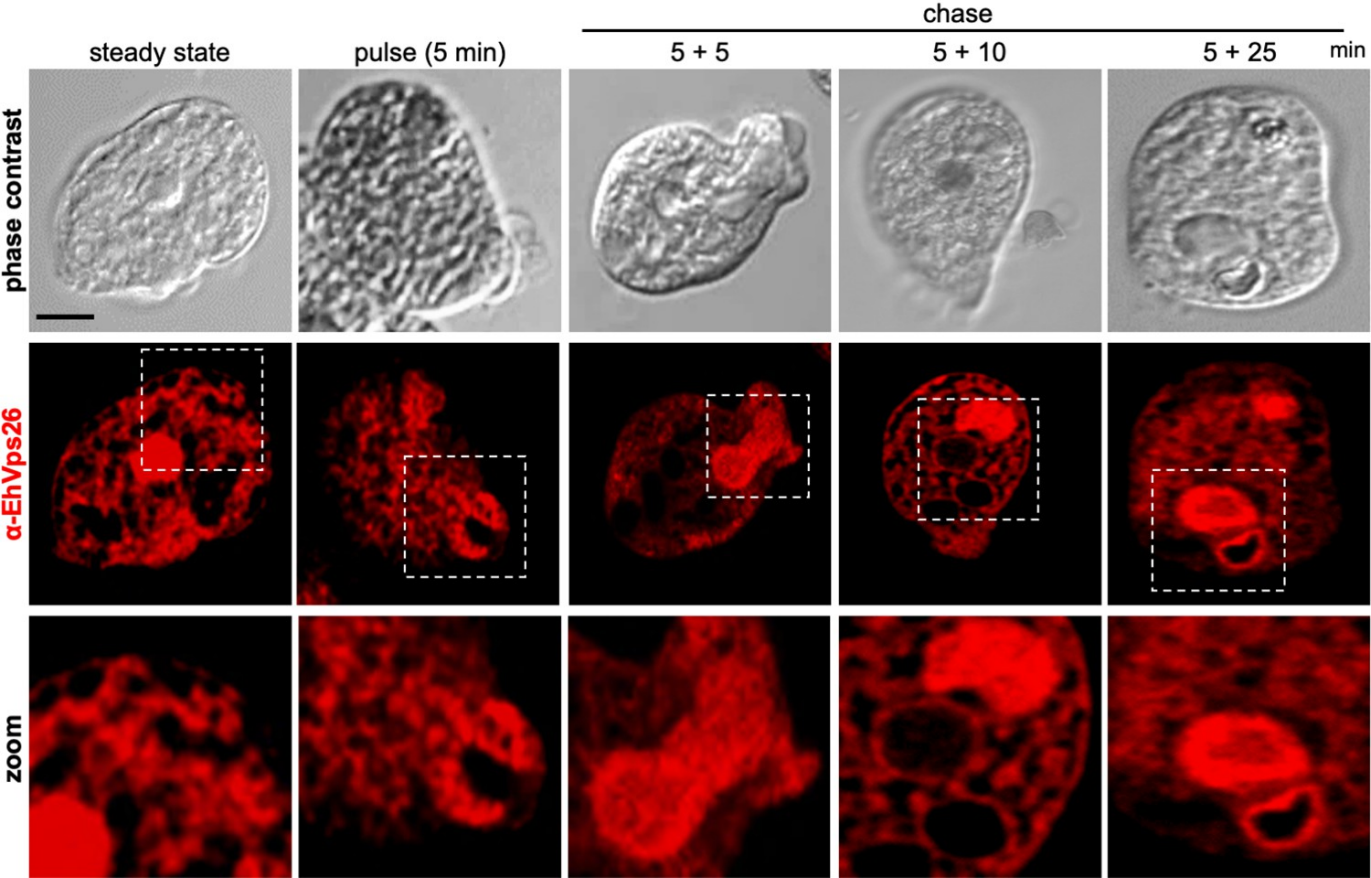
EhVps26 role during phagocytosis kinetics. Confocal microscopy using α-EhVps26 antibodies through the steady state, pulse (5 min), and pulse (5 min) and chase (5, 10, and 25 min) of phagocytosis. Squares at the bottom: Zoom of marked areas. Scale bar: 10 μm.

A recent proteomic analysis of phagosomes confirmed that EhVps26, EhVps35, and EhVps29 are present in the membrane proteins fraction [73]. Also, previous research detected EhVps26 in trogosomes and phagosomes, and it is involved in the transport of a cysteine protease receptor [48, 74]. We followed up this protein from the primary contact of trophozoites with target cells up to the internalization and degradation of the prey. Thus, we performed immunofluorescence experiments during pulse and chase phagocytosis assays to avoid the noise of recent red blood cells (RBCs) ingested (Fig 10). At 5 min (pulse) and 10 min (5 min pulse+5 min chase), we detected EhVps26 at the sites of trophozoites contact with RBCs, and in the phagocytic cups and channels. Later, at 15 min (5 min pulse+10 min chase) and 30 min (5 min pulse+25 min chase), EhVps26 was found upon and surrounding the RBCs-containing phagosomes, in many cases, close to the plasma membrane (Fig 10). These results evidenced the presence of the protein along the whole phagocytosis pathway, probably transporting virulence-involved molecules and other molecules. These findings strengthen the hypothesis of active cargo transport by retromeric proteins during phagocytosis, as demonstrated for EhVps35 [50]. Some of the molecules that have been involved in phagocytosis include EhRab1, EhRab7E, EhCP3, EhCP5, EhCP6, amoebapores, the Gal/GalNac lectin, lysozymes, EhADH, and the EhCPADH complex [75–77]. Therefore, some of them or their receptors could be retrieved by the retromer.

### 1.10 The *Ehvps26* gene knockdown affects the rate of phagocytosis in trophozoites

**Fig 11:**
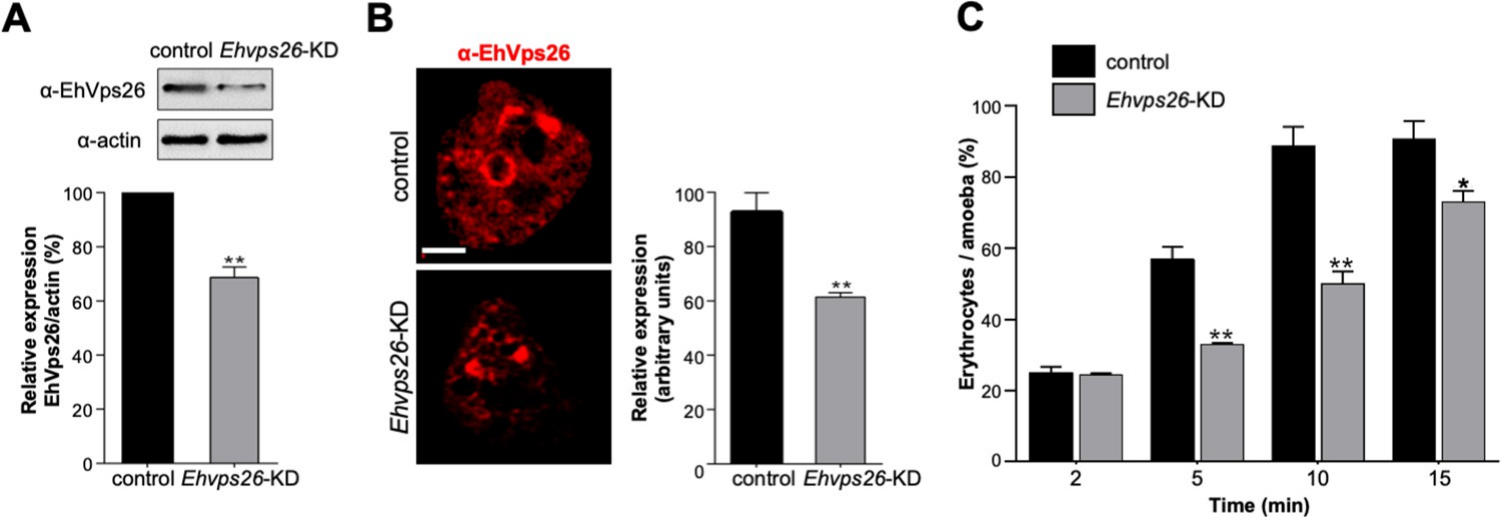
*Ehvps26* gene knocking down and its effect in trophozoites phagocytosis. **A.** Western blot of EhVps26 in control and *Ehvps26*-knocked down cells (*Ehvps26-*KD). Control: Trophozoites that were not fed with dsRNA. Bottom: Densitometry of the bands of three independent experiments using α-actin as a loading control. **B**. Confocal immunofluorescence of EhVps26 in control and *Ehvps26-*KD using α-EhVps26 antibodies. At right: Immunofluorescence quantification of at least 15 z-stacks of 0.5 μm from independent cells. **P<0.01. **C.** Rate of phagocytosis of control and *Ehvps26*-KD cells. The plot is representative of two independent experiments by triplicate. *P<0.05, **P<0.01.

To investigate the relevance of EhVps26 in phagocytosis, we knocked down the *Ehvps26* gene, using dsRNAs to partially inhibit gene expression [78], since no systems for knocking out genes have been established in *E*. *histolytica* yet. The mutant trophozoites (*Ehvps26*-KD) presented 30% less of EhVps26 protein, according to western blot assays (Fig 11A). Meanwhile, immunofluorescence experiments evidenced a reduction of 40% (Fig 11B). Afterward, we used the *Ehvps26*-KD trophozoites to measure the rate of phagocytosis versus the control cells (with no dsRNAs added in media). The knocked-down trophozoites ingested 50% fewer erythrocytes than the control cells (Fig 11C) at 5 and 10 min of interaction, whereas, at 15 min, the decrease in the rate of phagocytosis was 20%. The EhVps26 alteration triggers changes in the retromer complex, affecting the recycling process or other functions in which EhVps26 may be involved. Altogether, our findings strongly point out the importance of the EhVps26 protein in the chain of events orchestrating phagocytosis.

In conclusion, our results highlight the importance of the EhVps26 IDR in the protein structure stability, which also become significant for the interaction with known and yet unknown molecules, including cargoes. Additionally, our experimental data demonstrated that EhVps26 is a relevant protein during phagocytosis.

## Discussion

The interest of our group has been, for many years, the vesicular trafficking, phagocytosis and virulence of the *E*. *histolytica* protozoan parasite. Here, we first deepened our knowledge of the *E*. *histolytica* retromer by comparing its components among different organisms. The analyzed species conserve the CSC components, in charge of selecting cargoes, and, in some cases, they display more than one Vps26, Vps35, or Vps29 proteins. The main differences among them were found in the SNX membrane-deforming complex configuration (Fig 1A), responsible for endosomal phospholipids detection and membrane curvature, which suggests that this complex has been diversified depending on the organism needs [31]. Our bioinformatical analyses revealed that the *E*. *histolytica* retromer adopts a CSC-SNX3 configuration (Fig 1A) [49]. Accordingly, in the metazoan and fungal models, the CSC-SNX3 conformation allows tubules formation in the endosomal membrane, facilitating cargo recognition [27, 49, 52, 64, 79], which could occur in the same manner in the amoebic retromer.

Particularly, Vps26 proteins mediate the CSC assembly, allowing the interaction with cargo molecules [35]. According to the EhVps26 structural characterization, it is indeed a *bona fide* retromeric member: it possesses the Vps26 domain, which includes the EDCL and IMDGAPVKGESI equivalent motifs (Fig 1B and 1C), required for EhVps35 and EhSNX3 interaction, as described in mammalian orthologues [27, 33, 54]. In addition, a noticeable finding in EhVps26 was the presence of a peculiar long and highly charged IDR, enriched in Glu and Lys residues (Fig 1B), that provides flexibility and stability to the protein and promotes ligands binding [40]. The EhVps26 MDS predicted that under physiological conditions, the IDR remained unstructured, as described for other IDRs [80]. Further studies will be necessary to prove if the EhVps26 IDR adopts secondary structures under ligand stimuli. The use of a truncated version of EhVps26, lacking the IDR, confirmed the relevance of EhVps26 IDR in the structure and stability of the whole protein. The EhVps26-t 3D structure lacked the putative binding pockets for EhVps35 and EhSNX3 (Fig 5C), showing that the EhVps26 IDR is essential for the secondary structures stability. According to the RMSD, RMSF, Rg, and SASA parameters, the IDR provides high mobility to the EhVps26 protei [81]. High RMSD values have been observed by MDS in proteins with long IDRs [82, 83]. Additionally, the EhVps26 IDR flexibility may allow the protein to adopt different conformations, facilitating the interaction with other molecules. A recent work confirmed that a Vps35 mutated form, produces a Vps26 switch on its interaction region with the CSC, so, the protein may adopt a variety of poses, even when no SNX proteins are involved [64]; this could be also happening in EhVps26.

The AlphaFold2 server predicted that the components of CSC and EhVps26-EhVps35-EhSNX3 interact in a similar manner to the mammalian ones (Fig 6), reinforcing that a CSC-SNX3 configuration is functional in amoeba.

We detected EhVps26 at the trophozoites plasma membrane, a location probably mediated by post-translational modifications such as palmitoylation, myristoylation, and glycosylation, as we found more than 10 putative lipid and carbohydrates binding sites (S2 Table).

EhVps26 protein was also identified in the membranous network that corresponds to a Golgi-like organelle, according to the NBD-C6 ceramide marker, and TGN38 antibodies [71, 72].

We hypothesizd that EhVps26 may follow RBCs during the phagocytosis process, raising the question if the retromer starts the target proteins recycling since the first contact with the prey. Since several other proteins have been located in this pathway, we do not discard that EhVps26 forms functional complexes with some of them. The IDR can function as a linker, depending on the cargo [84]. EhVps26 could be transporting the proteins for RBCs targeting, engulfment and degradation, and for the recovery and recycling of molecules required by trophozoites for other physiological or pathological events.

The relevance of EhVps26 in phagocytosis was confirmed by *Ehvps26*-knocked down cells that lost 40% efficiency to ingesting RBCs (Fig 11B), probably caused by the impairment of cargo molecules recognition. Under this condition, the EhVps26 protein alteration may also distress the formation of the CSC, disturbing cargo proteins trafficking.

**Fig 12:**
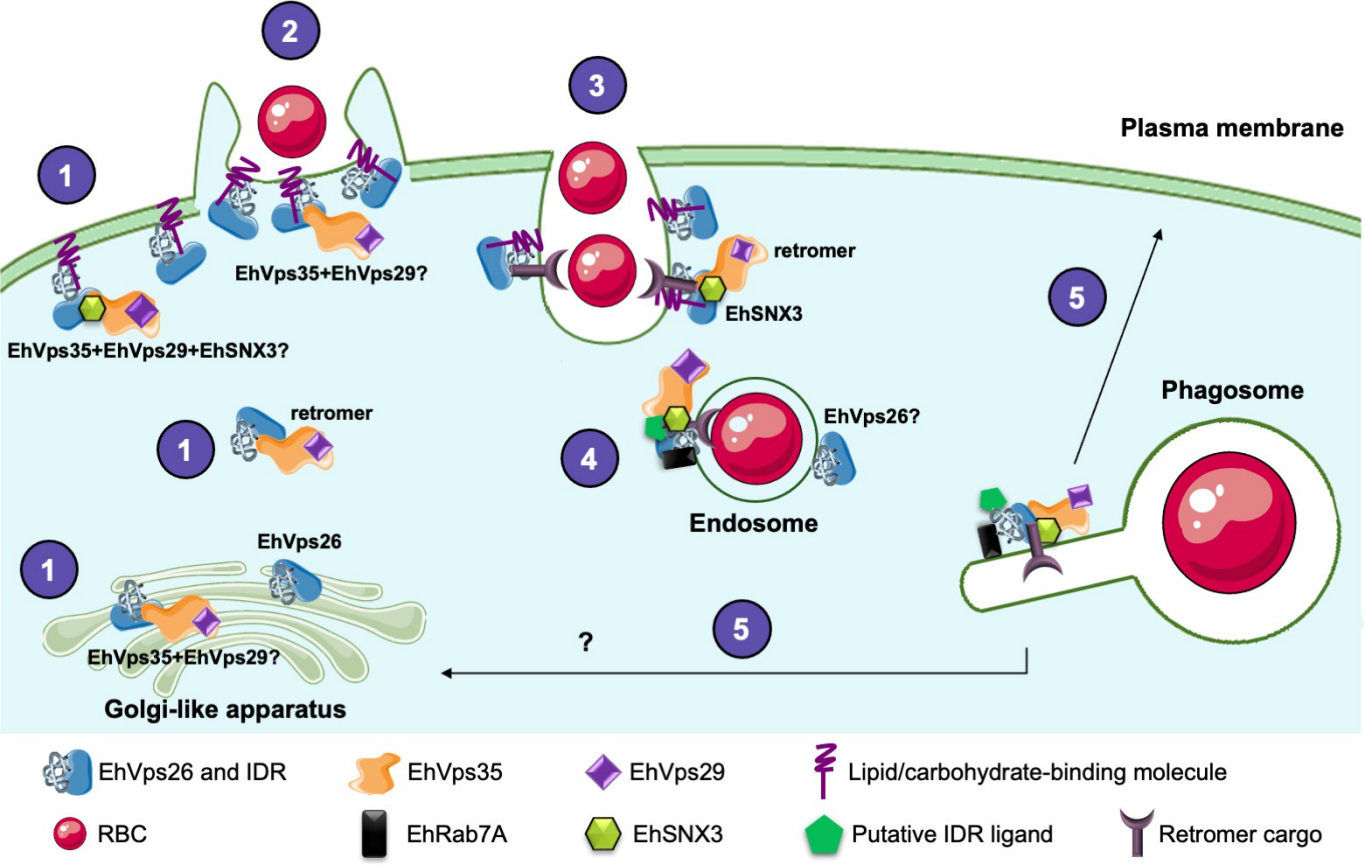
EhVps26 working model in *E*. *histolytica*. 1. Cellular localization of EhVps26 and the retromer at the plasma membrane, cytosol and Golgi-like apparatus. **2.** EhVps26, EhVps35 and EhSNX3 location in the phagocytic cups at the first contact with RBCs. **3.** EhVps26 and the retromer interacting with cargo molecules during RBCs engulfment in the phagocytic cup. **4.** EhVp26 and the retromer on the endosomal membrane. **5.** Cargo recycling to the plasma membrane or the Golgi-like apparatus-like.

According to previous results [48, 49], in steady state, the CSC is fully assembled at the amoebic cytosol (Fig 12). EhVps26 could be alone or forming part of the retromer at the plasma membrane and Golgi-like apparatus, however, further research will be necessary to elucidate this. When trophozoites enter in contact with target RBCs, EhVps26 is re-localized from plasma membrane to phagocytic cups and channels, probably selecting cargo molecules for their transport. As EhVps35 has been also found in in plasma membrane, cytoplasm, endosomes and phagosomes, it is possible that the retromer is already assembled at these sites. Once the phagosomes are formed, the retromer completes the cargo selection, then, according to our results, a pathway could be activated for transporting cargo molecules to the Golgi-like apparatus, facilitating their transit to target organelles. The putative cargo molecules for the retromer include the phagosome-resident proteins, but we also propose that, during the whole phagocytic process, the EhVps26 IDR might function as interaction site for other molecules.

In conclusion, the study of the retromer complexes and their components in protozoan organisms can give valuable clues for understanding the evolution of this structure. Additionally, the analysis of the EhVps26 IDR may provide information regarding other functions of this region in disease-causing organisms.

## Materials and methods

### Ethics statements

Cinvestav fulfills the standards of the Mexican Official Norm (NOM-062-ZOO-1999) “Technical Specifications for the Care and Use of Laboratory Animals”, based on the Guide for the Care and Use of Laboratory Animals (“The Guide” 2011, NRC, USA, with the Federal Register Number BOO.02.03.02.01.908), awarded by the National Service for Agrifood Health, Safety and Quality (SENASICA). This organization verifies the state of compliance of such NOM in México and belongs to the Ministry of Agriculture and Rural Development. The Institutional Committee for Animal Care and Use (IACUC/Ethics committee) from Cinvestav, the regulatory office for research protocols’ approval involving the use of laboratory animals, reviewed and approved all animal experiments (Protocol Number 0505–12, CICUAL 001).

### Sequences analysis

We retrieved the Vps26, Vps35, Vps29, SNX1, SNX2, SNX3, SNX5 and SNX6 amino acid sequences of *Homo sapiens* and *Mus musculus* from the NCBI protein database, meanwhile, the sequences from *E*. *histolytica*, *E*. *dispar*, *E*. *invadens*, *N*. *fowleri*, *P*. *falciparum* and *T*. *vaginalis* were obtained through BLASTp analysis in the VEuPathDB servers [85], using the human sequences as reference (access numbers in S1 Table). We considered experimentally proved proteins, isoforms and non-experimentally proved sequences. To analyze the domains, we used the server InterPro [86]. The prediction of IDR was made with the servers Disopred3 [87], IUPRED2A [88, 89], and NetSurfP-2.0 [90]. Results were graphed with IBS software [91], while the post-translational modifications (PTM) in EhVps2 were predicted with algorithms archived in the ExPasy server (expasy.org) The sequences were aligned by the Clustal W algorithm in the T-coffee server [92] and the results were visualized in the Jalview software [93, 94].

### Tridimensional models

To determine the EhVps26 amoebic protein tridimensional structure, we used servers based on different prediction methods, such as threading (I-tasser [95–97]), homology (SWISS-MODEL, [98]; RaptorX, [99]; Modeller 10.2 [100]; and Phyre2 [101] and *ab initio* [102]. The obtained models were analyzed by Ramachandran plot in the Molprobity server [103]. The chosen model was refined through the GalaxyWEB server [104] and analyzed again with Molprobity. The structure was aligned with human and mouse orthologs (PDB ID: 2FAU [33]; PDB ID: 2R51 [32] by the use of the MatchMaker tool in Chimera software [105], where RMSD values were taken as a reference for structural identity. We created an additional tridimensional model corresponding to the first 300 amino acids of EhVps26, lacking the -C terminal. All the images were obtained with Chimera.

### Molecular dynamics simulations

Tridimensional structures corresponding to full EhVps26 and EhVps26 lacking C-terminal (EhVps26-t) were prepared by the CHARMM-GUI server [106] using the CHARMM36m forcefield [107]. The system for EhVps26 was solvated with 169083 water molecules and equilibrated by adding 483 and 498 chloride and potassium ions. Meanwhile, for EhVps26-t, we used 126 Cl and 126 K ions for equilibration and 44806 water molecules for solvation. The systems solvation was based on TIP3 water to generate a cubic box with a 10 Å margin which allowed the molecules to move through the simulations. The systems were minimized and equilibrated during 10,000 and then 250,000 steps, under an NVT ensemble (constant particle number, volume, and temperature) at 310 K for 1 nanosecond with protein atoms restrained. The files generated from equilibration steps were used to run a 500 ns lasting MDS, under an NPT ensemble (constant particle number, pressure, and temperature), where the temperature was maintained constant by the use of Langevin dynamics, and stabilized at 1 bar pressure [108, 109]. EhVps26 and EhVps26-t proteins were considered soluble, without position restraints, and under periodic boundary conditions. A timestep of 2.0 fs was used and coordinates were saved for every one ps. MDS were run with NAMD 2.12 software (http://www.ks.uiuc.edu/Research/namd/, [110]) at Hybrid Cluster Xiuhcoatl (http://clusterhibrido.cinvestav.mx) of CINVESTAV-IPN, México. The MDS trajectory was analyzed by the Carma Software [111]. A previously reported script [112] was used in the VMD software to calculate the solvent-accessible surface area (SASA). The root mean square deviation (RMSD), root mean square fluctuation (RMSF), radius of gyration, and SASA were plotted with Microsoft Excel software. The secondary structure prediction (DSSP) was calculated with the Timeline plugin in VMD.

### Binding pockets analysis

To predict potential binding pockets, the average equilibrated structures of EhVps26 and EhVps26-t were submitted to the DOGSiteScorer [60] server. We chose the sites with higher *druggability* scores, which means a large volume, high depth, high polar amino acids amount, and sequence location. The validation of binding pockets was calculated with the Protein Allosteric Sites Server (PASSer, [113, 114]). Sites were visualized with Chimera.

### Tridimensional prediction of amoebic CSC and CSC-SNX3 complexes

The amino acid sequences of EhVps26 (EHI_062490), EhVps35 (EHI_002990), EhVps29 (EHI_025270) and EhSNX3 (EHI_004400) were used as input in AlphaFold2 algorithm [65]. Multimer option and three models per prediction were set in the Cosmic^2^ server [66]. We chose the models with higher confidence scores, which were compared with mouse and human crystals, respectively [27, 76]. Images were obtained with Chimera and interacting residues were predicted with the PDBSum server [115].

### *E*. *histolytica* cultures

*E*. *histolytica* trophozoites (strain HM1:IMSS) were axenically cultured in TYI-S-33 medium [116] that was supplemented with adult bovine serum (ABS) (Equitech-Bio, Inc.) at 37°C. For experiments, the amoebae were harvested at the logarithmic growth phase as described [117].

### Constructs generation

Amoebic genomic DNA was obtained using the kit Wizard® Genomic DNA Purification Kit (Promega, TM050) following the manufacturer’s instructions. For the histidine-tagged recombinant protein, the full ORF of the *Ehvps26* gene (EHI_062490) was amplified by PCR using the primers: sense GGTAC’CATGGCTTTCTTATTTGGTACAC and antisense G’GATCCTTAAAATAAGTTGTCATCATCTTGTTTA where the bold letters indicate the nucleotides sequences for *KpnI* and *BamHI* endonucleases recognition sites. For the knockdown experiments, the first 400 pair bases of the *Ehvps26* gene were amplified with the primers: GAGCT’CATGCTTGTACTTGTTATTGGAGA (sense) and GGTAC’CTTTTATTAACTGACACTCTTAAAT (antisense). The bold characters correspond to recognition sites for *SacI* and *KpnI* endonucleases. The genes were cloned in the pJet1.2/blunt vector (ThermoFisher) and subcloned in the corresponding sites of the pCold I (Takara Bio, Inc.) and L4440 vectors (Addgene). Constructs were named pCold-*Ehvps26* and pL4440-*Ehvps26*, respectively. The recombinant protein had a His-tag in the N-terminal.

### Generation of α-EhVps26 polyclonal antibodies

*Escherichia coli* BL21 (DE3) bacteria were transformed with the pCold-*Ehvps26* construct. The recombinant protein production was induced at 16°C using of 100 μM isopropyl- β-thiogalactoside (IPTG) to the media culture. The recombinant protein (EhVps26r) was purified with the HisPur Cobalt Resin (Thermo Scientific), following the manufacturer’s instructions. To produce the antibodies, an initial dose (300 μg) of EhVps26r was mixed with Titer-Max Gold (Sigma-Aldrich) (v:v), and subcutaneously and intramuscularly inoculated in a male New Zealand rabbit. The rabbit was housed in the Cinvestav vivarium and monitored for clinical signs of disease until the end of inoculations. Three additional doses of 100 μg of EhVps26r were subcutaneously administered at intervals of 14 days. Serum was obtained before each immunization. For the euthanasia, the rabbit was humanely anesthetized with an intravenous dose of tiletamine and zolazepam (Zoletil) and bled from the auricular artery.

### Western blot assays

Logarithmic phase *E*. *histolytica* amoebae were lysed by thermal shock in liquid nitrogen and proteases inhibitors (PMSF 100 mM, benzamidine 100 mM, aprotinin 10 mg/ml, pepstatin 1mg/ml, leupeptin 10 mg/ml and E-64 1 mg/ml). 35 μg of total extracts (TE) were separated in 12% SDS-PAGE, transferred to nitrocellulose filters, and probed with α-EhVps26 polyclonal rabbit (1:1000) or with monoclonal mouse α-histidine (1:500, Abcam) or mouse α-actin (1:1000) (kindly donated by Dr. Manuel Hernández-Cinvestav) antibodies. Membranes were incubated with primary antibodies at 4°C overnight, then they were washed with PBS Tween 0.01% and incubated with the respective α-rat or α-mouse-HRP antibodies (Zymed, 1:10,000). The signals were developed with ECL Prime western blotting detection reagent (G&E-healthcare) Each experiment was performed by triplicate.

### Confocal microscopy

Amoebae were grown on glass coverslips, fixed with 4% paraformaldehyde, and permeabilized with 0.5% Triton X-100. The samples were blocked with 10% PBS-BSA and incubated for 1 h with rabbit α-EhVps26 (1:100), and mouse α-EhMRE11 (1:100, Santa Cruz, USA) antibodies at 37°C. Samples were washed and incubated with α-rabbit antibodies coupled to Cy5 (1:200, Thermo Fisher) or α-mouse coupled to FITC (1:200, Thermo Fisher). Fixed samples were exhaustively washed, mounted using VECTASHIELD (Vector Labs), and visualized through a Carl Zeiss LSM 700 confocal microscope, considering 0.5 μm z-stacks. The Golgi-like organelle was detected using the NBD-C6-ceramide probe (Thermo Fisher), and mouse α-TGN38 (1:100, Santa Cruz, USA) antibodies, following the manufacturer’s instructions. Images were processed with the Zen Black software (Carl Zeiss, 2012). We also used the colocalization module within the Zen Blue software (Carl Zeiss, 2012).

### Transmission electron microscopy

Trophozoites in basal conditions were fixed with 0.5% glutaraldehyde and 4% paraformaldehyde in PBS for 1 h at room temperature, washed with PBS and dehydrated with increasing concentrations of ethanol. Samples preparation was followed as reported by others [72]. After infiltration, samples were embedded in LR White resin (London Resin Co., London, UK) and polymerized under UV at 4°C for 24 h. Thin sections (60 nm) were mounted on Formvar-coated nickel grids followed by incubation overnight at 4°C with rabbit α-EhVps26 and mouse α-TGN38 antibodies (1:40). Then, grids were washed and incubated for 1 h with a 30 nm gold-conjugated anti-rabbit and 15 nm gold-conjugated anti-mouse antibodies (1:50) (TED Pella Inc., Redding, CA, USA) respectively. Sections were contrasted with uranyl acetate and lead citrate for analysis through a Jeol-1400 transmission electron microscope (JEOL Ltd. Tokyo, Japan). Slides incubated only with secondary antibody were used as negative control.

### Phagocytosis assays

Peripheral human red blood cells (RBCs) were added to amoebic cultures in a ratio of 1:25 (amoeba:erythrocyte) for 5 min as the pulse time. Then the RBCs were lysed by the addition of sterile water and samples were incubated for 5, 10, 15, or 30 min in TYI medium at 37°C to chase the ingested RBCs. After each time, trophozoites were fixed and managed for immunofluorescence, as described above. Other samples were processed by the Novikoff technique [118] to visualize and quantify the RBCs phagocytosed per amoeba.

### *Ehvps26* gene knocking down in trophozoites

We used the methodology reported by Solis et al., 2009 [78] to knock down the *Ehvps26* gene. Briefly, we transformed RNAse III-deficient *E*. *coli* HT 115 bacteria (rnc14::ΔTn10) with pL4440-*Ehvps26* construct. The bacteria were grown in LB agar supplemented with 0.1 mM ampicillin and 0.001 mM tetracycline. Positive colonies were verified by PCR. The production of dsRNA was induced by the addition of IPTG to the culture media and after the bacteria lysis, the remaining dsRNA and ssRNA were removed by adding DNAse I (Invitrogen) and RNAse A (Ambion) to samples. To induce the *Ehvps26* gene knockdown, purified dsRNA was added to the amoebic culture (1x10^5^) at a final concentration of 10 μg/ml in TYI-S-33 medium. after 96 h, the knockdown level was verified by Western blot and immunofluorescence, as previously described. Amoebae grown without added dsRNA were used as a control.

### Data analysis and statistical methods

The values of the experiments are expressed as mean ± standard error of three independent experiments. Plots and statistical analysis were made using GraphPad Prism version 6.0 for Windows, GraphPad Software, La Jolla California USA. For statistical analysis, we used unpaired t-student tests. P values are described in each figure.

## Supporting information

S1 Fig Ramachandran plots of predicted EhVps26 3D models. Plots were obtained by different software: **a**. i-Tasser, **b.** SWISS-MODEL, **c.** RaptorX, **d.** Modeller, **e.** Phyre2, **f.** Robetta.
(TIF)

S1 Table Identity among EhVps26 and Vps26 orthologues. (TIF)

S2 Table Membrane anchoring-related post-translational modifications in EhVps26. Underlined residues point out to consensus post-translational modification sites.
(TIF)

S1 Raw images (PDF)
