# Supplementary figures and images for: The Entamoeba histolytica Vps26 (EhVps26) retromeric protein is involved in phagocytosis: Bioinformatic and experimental approaches
Source: PLoS One. 2024 Aug 8;19(8):e0304842. doi: 10.1371/journal.pone.0304842 (PMC11309391; doi:10.1371/journal.pone.0304842)

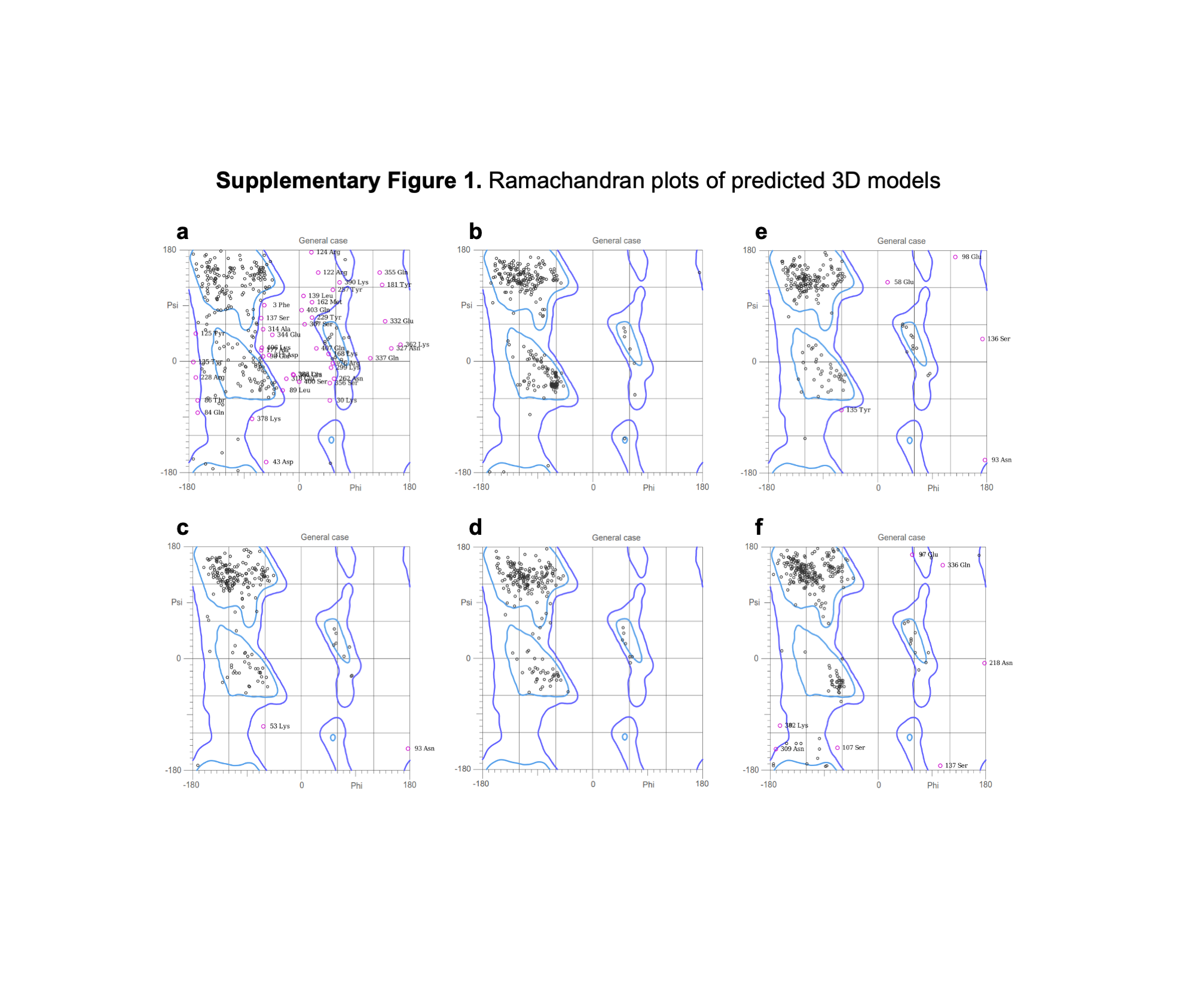

Supplement: S1 Fig — Plots were obtained by different software: a. i-Tasser, b. SWISS-MODEL, c. RaptorX, d. Modeller, e. Phyre2, f. Robetta. (TIF) [file pone.0304842.s001.tif]

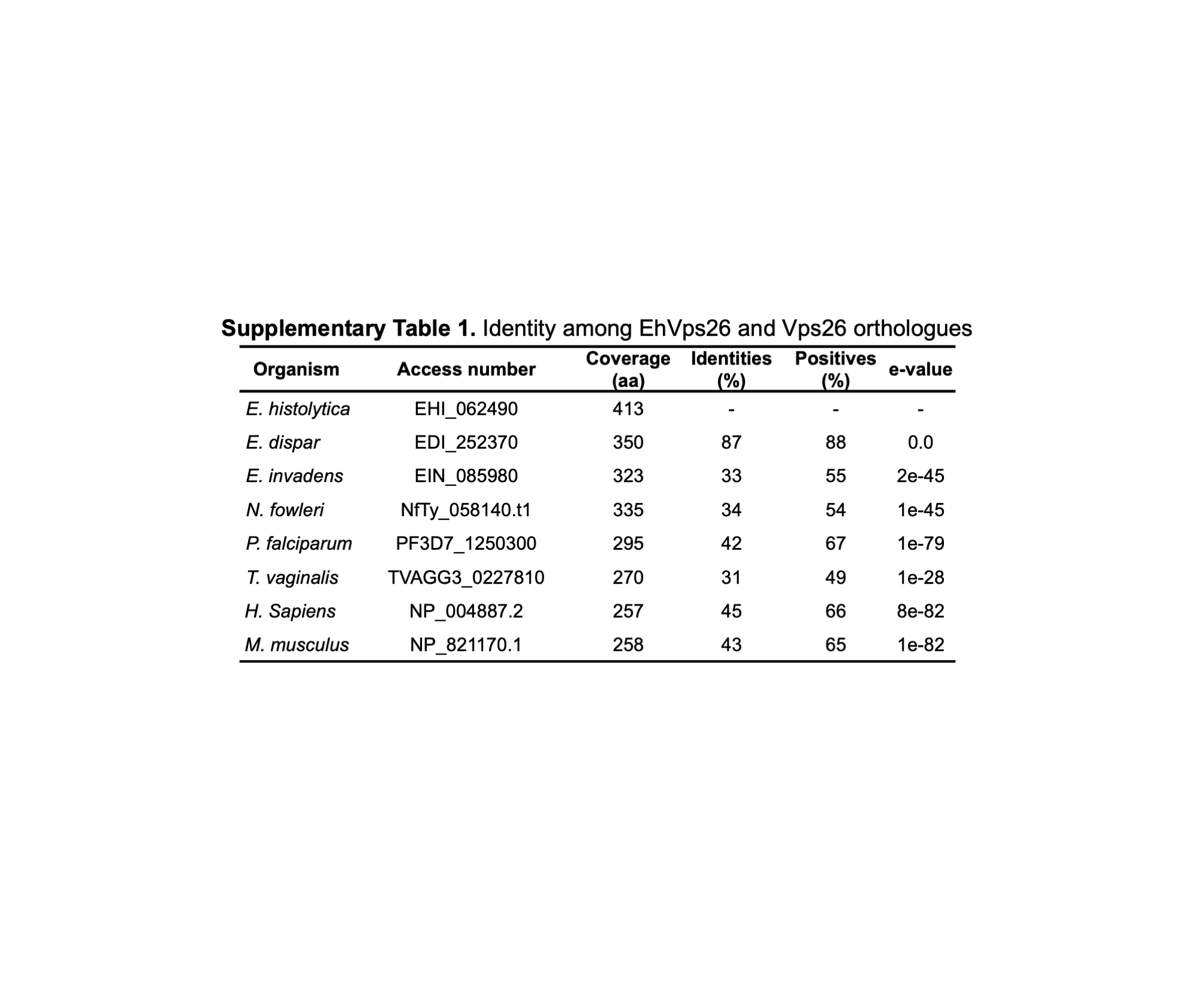

Supplement: S1 Table — (TIF) [file pone.0304842.s002.tif]

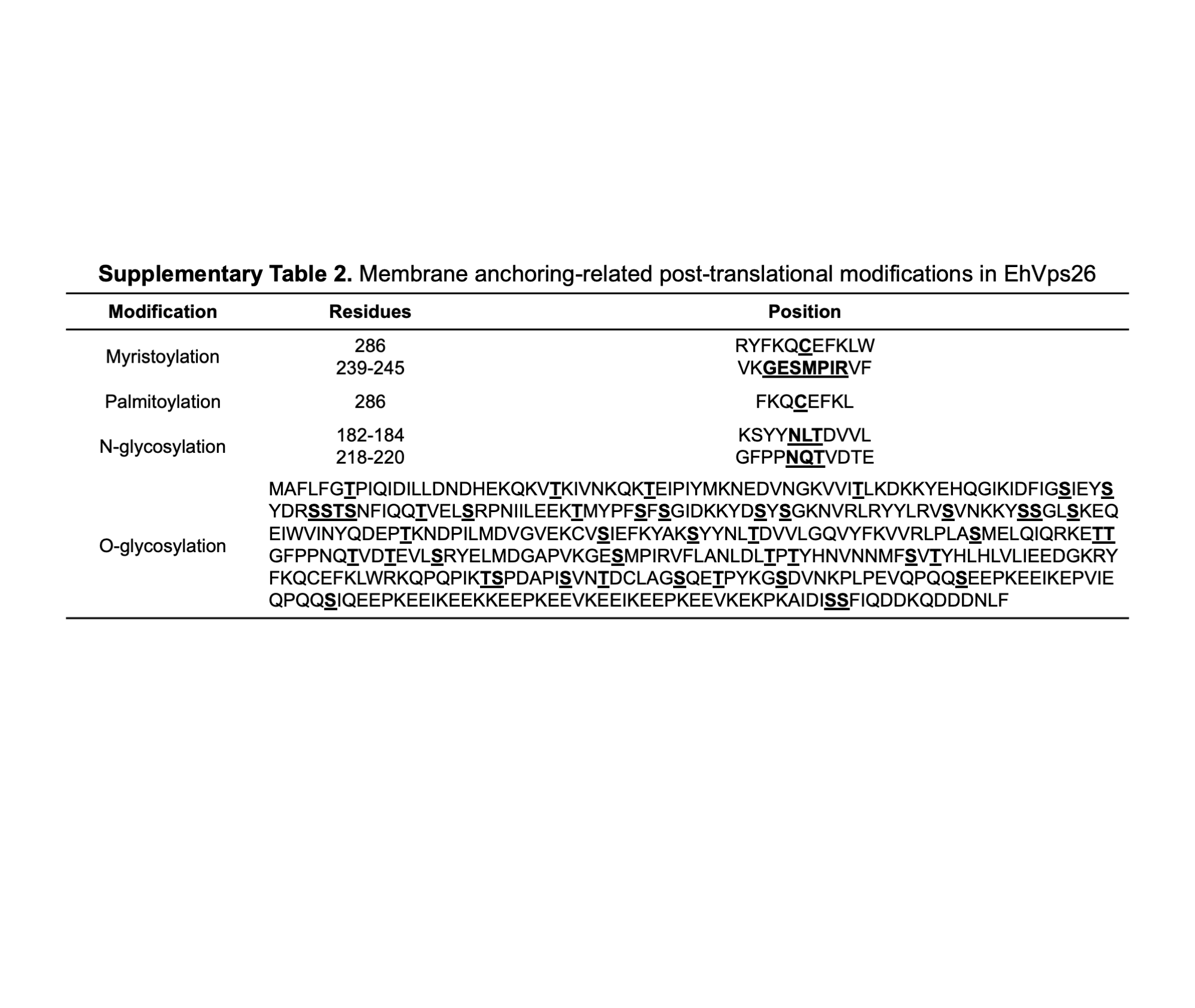

Supplement: S2 Table — Underlined residues point out to consensus post-translational modification sites. (TIF) [file pone.0304842.s003.tif]

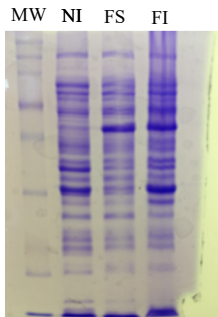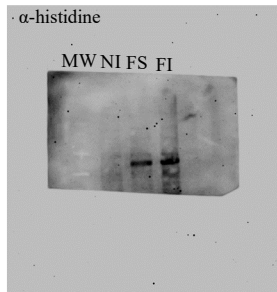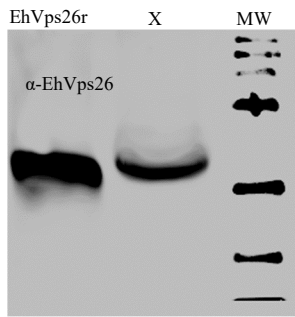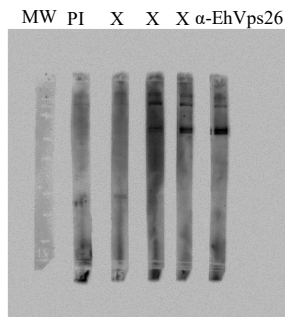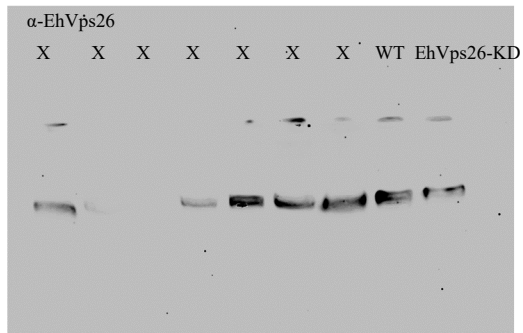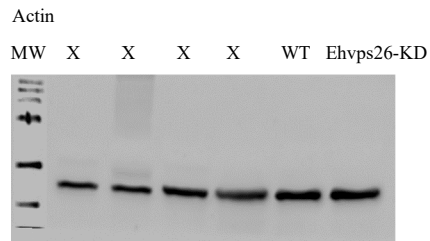

Supplement: S1 Raw images — (PDF) [file pone.0304842.s004.pdf]
